# Multitask Artificial Intelligence–Based Electrocardiogram Tool for Preoperative Cardiac Testing in Noncardiac Surgery: Retrospective Cohort Study of Health Care Utilization and Costs

**DOI:** 10.2196/90099

**Published:** 2026-06-17

**Authors:** Hong-Mi Choi, Yerin Kim, Joonghee Kim, Jiesuck Park, In-Chang Hwang, Yun Young Choi, Ji Hyun Lee, Yeonyee E Yoon, Il-Young Oh, Goo-Yeong Cho, In-Ae Song, Youngjin Cho

**Affiliations:** 1 Department of Cardiology, Cardiovascular Center Seoul National University Bundang Hospital Seongnam, Gyeonggi Republic of Korea; 2 Department of Internal Medicine College of Medicine Seoul National University Seoul Republic of Korea; 3 Department of Emergency Medicine Seoul National University Bundang Hospital Seongnam, Gyeonggi Republic of Korea; 4 ARPI Inc Seongnam, Gyeonggi Republic of Korea; 5 Department of Anesthesiology and Pain Medicine Seoul National University Bundang Hospital Seongnam, Gyeonggi Republic of Korea; 6 Department of Anesthesiology and Pain Medicine College of Medicine Seoul National University Seoul Republic of Korea

**Keywords:** artificial intelligence, electrocardiography, perioperative care, mortality, prediction, preoperative cardiovascular examination, cost

## Abstract

**Background:**

Preoperative cardiovascular risk stratification is essential in noncardiac surgery, but conventional testing is frequently overused, increasing costs without improving outcomes. Artificial intelligence (AI)–enabled electrocardiography (ECG) may enhance perioperative risk assessment by identifying surgical candidates at very low-risk for adverse events.

**Objective:**

This study aimed to evaluate whether AI-ECG–based risk stratification could help decrease low-yield preoperative cardiovascular testing and reduce associated costs, without an observed increase in postoperative adverse outcomes, in candidates for noncardiac surgery.

**Methods:**

We retrospectively analyzed 41,218 patients (46,135 ECG-surgery pairs) undergoing noncardiac surgery at Seoul National University Bundang Hospital (2020-2021). An AI-ECG algorithm generated eight probability scores for cardiac conditions, classifying cases as low- or high-risk. Based on the performance and results of preoperative cardiovascular testing (transthoracic echocardiography, coronary computed tomography angiography, single-photon emission computed tomography, or coronary angiography), cases were classified as no advanced cardiovascular imaging, negative-test, or positive-test. The primary end point was a 30-day composite of all-cause mortality and unplanned percutaneous coronary intervention.

**Results:**

AI-ECG classified 92.3% (42,599/46,135) of cases as low-risk, with a composite outcome rate of 0.2% (79/42,599) vs 2.9% (101/3536) in high-risk cases. Preoperative cardiovascular testing was performed in 11.8% (5458/46,135) of cases, with only 16.3% (892/5458) yielding positive findings. In AI-ECG low-risk cases, event rates were uniformly low (0.2%-0.4%) irrespective of whether advanced cardiovascular testing was performed, whereas in high-risk cases, rates were consistently high (2.6%-3.4%). The incidence of the composite outcome was consistently higher in AI-ECG–graded high-risk cases across all European Society of Cardiology surgical risk and Revised Cardiac Risk Index strata.

**Conclusions:**

In this retrospective cohort, a multitask AI-ECG identified surgical candidates at low-risk for postoperative complications, for whom advanced cardiovascular testing demonstrated low diagnostic yield. Integrating AI-ECG with conventional risk tools may offer an exploratory strategy to optimize resource use and minimize redundant testing. Prospective studies are needed to confirm the clinical and economic benefits of AI-ECG as a screening tool.

## Introduction

More than 200 million noncardiac surgeries are conducted worldwide each year [1], with numbers steadily increasing [2]. Preoperative cardiovascular testing imposes a major health care burden, with estimated costs of US $18 billion annually in the United States [3]. The substantially higher morbidity and mortality risks of postoperative cardiovascular complications underscore the importance of accurate preoperative risk stratification [4]. Although existing risk-estimation tools provide general estimates of perioperative cardiovascular risk [5,6], they do not specify whether additional testing is warranted and do not eliminate the risk of adverse outcomes. Despite current guidelines outlining which patients may benefit from advanced cardiovascular testing [5,6], in clinical practice, the demand for such testing often exceeds the recommended indications [7]. As a result, preoperative cardiovascular tests are frequently overused and thereby hinder efficient health care resource usage.

Electrocardiography (ECG) is a well-established low-cost preoperative assessment tool, but has limited utility in noncardiac surgery [3,8,9]. Not surprisingly, the quality of ECG-derived information has been highly dependent on the physician’s expertise and the patient’s characteristics [10,11]. Thus, recent guidelines have recommended ECG use in only selected patients with suspected cardiovascular diseases or risk factors who are undergoing intermediate- or high-risk noncardiac surgery [5,6]. However, recent advances in artificial intelligence (AI) have expanded the diagnostic and prognostic applications of ECG, enabling the detection of coronary artery disease, valvular heart disease (VHD), and myocardial dysfunction [12,13]. With its low-cost and broad accessibility, AI-enhanced ECG is a promising tool for preoperative risk stratification.

Building on a preexisting AI-ECG model that incorporated eight quantitative scores for electrocardiography (QCGs) reflecting diverse cardiac conditions [14-19], we aimed to evaluate whether AI-ECG–based risk stratification could help decrease low-yield preoperative cardiovascular testing and reduce associated costs, without an observed increase in postoperative adverse outcomes in cases of noncardiac surgery. We hypothesized that the integration of AI-ECG into the workflow of preoperative risk assessment could help identify a subgroup of cases with an extremely low-risk of postoperative adverse outcomes, thereby requiring no additional testing.

## Methods

### Study Population

This single-center, retrospective cohort study included cases of noncardiac surgery at Seoul National University Bundang Hospital between 2020 and 2021, and underwent an ECG within the 30 days preceding the surgery. The exclusion criteria included the following: (1) no preoperative ECG within 30 days preceding the surgery, (2) elective cardiac surgery or extracorporeal membrane oxygenation-related surgery, (3) the use of simple pain-control procedures, (4) surgeries performed under local anesthesia, (5) surgery for a deceased organ donor, (6) rigid bronchoscopy, (7) dental surgery, and (8) multiple surgeries during the index admission. A total of 46,135 ECG–surgery pairs from 41,218 patients were analyzed (Figure S1 in Multimedia Appendix 1). As this was a retrospective study, the sample size was determined by available data meeting the inclusion criteria during the specified study period.

### Data Collection

All ECGs were recorded under standardized settings (25 mm/s speed and 10 mm/mV gain) and processed to extract the QCG scores from the JPEG file. The preoperative ECG that was recorded within 30 days before the surgery and closest to the surgery was paired with one noncardiac surgery. For 26 patients who underwent coronary revascularization following preoperative cardiovascular evaluation, prerevascularization ECGs were specifically selected as the index ECGs. Consequently, these cases were exempted from the standard 30-day ECG inclusion criterion. This exception was made because using postintervention ECGs for the primary cohort assignment could introduce treatment bias and falsely obscure the utility of advanced cardiovascular testing. The treating clinicians were unaware of the retrospective AI-ECG risk scores, as the tool was not yet available at the time of surgery. Clinical data, including surgery-related records, test results, and outcomes, were obtained from electronic health care records. This observational study conformed to the STROBE (Strengthening the Reporting of Observational Studies in Epidemiology) reporting guideline [20].

### Ethical Considerations

This study’s protocol was approved by the Institutional Review Board of Seoul National University Bundang Hospital (IRB No. B-2409-927-106), which waived the requirement for written informed consent owing to the retrospective nature of this study. All data were deidentified, and no financial compensation was provided to study participants. All clinical investigations were conducted in accordance with the principles of the Declaration of Helsinki.

### AI Algorithm and Risk-Stratification Strategy for AI-ECG

The QCG system is a deep-learning–based AI analyzer that uses 12-lead ECG images (in JPEG, PNG, or PDF formats) as input data and yields the probability score of specific downstream tasks as numerical values (range 0-100). Following pretraining using self-supervised learning schemes, these models were fine-tuned using multitask learning schemes with a modified convolutional neural network using residual connections, squeeze excitation modules, and a nonlocal block. The input data comprised 47,194 annotated ECG images from Seoul National University Bundang Hospital that were recorded between 2017 and 2019. ECGs obtained during this study’s period (2020-2021) were not used for model training, fine-tuning, or calibration and were reserved exclusively for outcome analysis. The tasks included rhythm classification and the construction of ten QCG scores related to various medical emergencies: (1) critical illness (critical score), (2) acute coronary syndrome (ACS), (3) ST-segment elevation myocardial infarction, (4) myocardial injury, (5) pulmonary edema, (6) pericardial effusion, (7) left ventricular dysfunction, (8) right ventricular dysfunction, (9) pulmonary hypertension, and (10) hyperkalemia. ECG buddy—an app that provides these 10 QCG scores—was approved by the Korean Ministry of Food and Drug Safety (January 2024). Detailed explanations of the AI algorithms and validation studies have been previously published [15-18]. For this dataset, the QCG scores were generated using version 1.0.6 of the app. Thresholds and clinical interpretations of each QCG score are provided in Table S1 in Multimedia Appendix 1.

Among the ten probability scores, we selected eight QCG scores that are directly related to the cardiac conditions of interest, except for the critical and hyperkalemia scores. All scores had their own optimal thresholds to maximize diagnostic performance, which were determined at the model-development stage. The participants were assigned to the AI-ECG low-risk group when all 8 QCG scores were lower than their individual optimal thresholds; otherwise, they were assigned to the AI-ECG high-risk group.

### Preoperative Advanced Cardiovascular Imaging

Preoperative advanced cardiovascular imaging included echocardiography, coronary computed tomography angiography (CCTA), single-photon emission computed tomography (SPECT) performed within 90 days before surgery, and coronary angiography (CAG) performed within 180 days before surgery. The decision for further cardiovascular testing and the choice of diagnostic modality were made by the attending surgeon or consulting physician while considering each patient’s functional capacity, individual risk, and surgical risk. Even if tests were included in routine clinical practice irrespective of the surgery, all cardiovascular tests performed within the designated time window were considered preoperative cardiovascular testing.

Positive findings of preoperative cardiovascular tests were selected based on widely accepted actionability, whereby positive results suggested the need for further preoperative evaluation. For echocardiography, positive findings were defined as the presence of any of the following: moderate or severe VHD (aortic stenosis or regurgitation, mitral stenosis or regurgitation, or tricuspid regurgitation); left ventricular ejection fraction ≤50%; or regional wall motion abnormality. For CCTA and CAG, ≥50% stenosis in any of the three major coronary arteries was considered a positive result; angiographic stenosis grades were used for CAG. For SPECT, a ≥10% reversible or irreversible perfusion defect was defined as a positive result. The costs of CCTA, CAG, and SPECT were obtained from a published study conducted in the Republic of Korea in 2018 that reported values in US dollars [21]. For echocardiography, the cost was based on the median value of publicly posted prices in Seoul, Republic of Korea, and was converted from Korean won to US dollars using an approximate 2021 exchange rate [22]. The exact per-test unit costs applied in this study were US $267 for echocardiography, US $232 for CCTA, US $485 for SPECT, and US $511 for CAG.

Based on the preoperative cardiovascular testing status, all the surgery-ECG pairs were categorized into three groups: no advanced cardiovascular imaging, cases without any preoperative cardiovascular test; negative test, cases that underwent testing without any positive findings; and positive test, cases with at least one positive finding. In this context, a negative test indicates the absence of predefined positive findings in a specific examination rather than the confirmation of overall clinical normality.

### Study Group Definition

**Figure 1:**
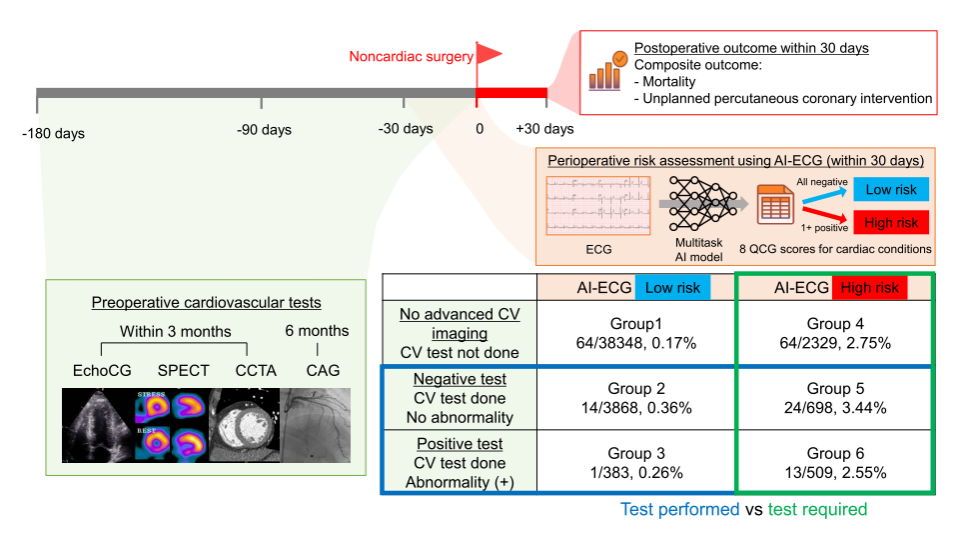
Study design. Among the preoperative CV tests, we included EchoCG, SPECT, and CCTA that were performed within 90 days, and CAG performed within 180 days before the noncardiac surgery. Among the ECG scans performed within 30 days preoperatively, the ECG obtained nearest to the surgery was selected to estimate the QCG scores using an AI-ECG. Next, the preoperative risk was classified using eight QCG scores related to cardiac conditions. Six groups were created based on the preoperative CV test and AI-ECG risk classification. The blue square denotes the surgeries in which CV tests were performed (groups 2, 3, 5, and 6; blue square), and the green square implies the surgeries in which testing would have been required (groups 4, 5, and 6; green square) assuming that the decision to perform preoperative CV testing strictly followed the AI-ECG risk stratification (ie, tests performed only in high-risk cases). AI-ECG: artificial intelligence–enabled electrocardiography; CAG: coronary angiography; CCTA: coronary computed tomography angiography; CV: cardiovascular; ECG: electrocardiography; EchoCG: echocardiography; QCG: quantitative score for electrocardiography; SPECT: single-photon emission computed tomography.

To compare the adequacy of the preoperative cardiovascular testing and AI-ECG, this study’s population was further divided into six groups: (1) group 1, low-risk AI-ECG and no advanced cardiovascular imaging; (2) group 2, low-risk AI-ECG and negative test; (3) group 3, low-risk AI-ECG and positive test; (4) group 4, high-risk AI-ECG and no advanced cardiovascular imaging; (5) group 5, high-risk AI-ECG and negative test; and (6) group 6, high-risk AI-ECG and positive test (Figure 1).

### Postoperative Outcome Definition

The primary measure that was used to assess the predictive performance of preoperative cardiovascular testing and AI-ECG was a composite postoperative outcome occurring within 30 days after the index surgery that included all-cause mortality and unplanned percutaneous coronary intervention (PCI). Mortality was restricted to deaths during hospitalization, including deaths within 30 days of readmission, whereas deaths that occurred outside the hospital were not included in the analysis. Given the substantial impact of cardiovascular comorbidities on postoperative critical care requirements, an exploratory composite outcome that incorporates prolonged mechanical ventilation for ≥3 days into the composite postoperative outcome was also analyzed. The overall study scheme is illustrated in Figure 1.

### Statistical Analysis

Continuous variables were summarized as means with SDs or medians with IQRs, whereas categorical variables were summarized as frequencies with percentages. Between-group comparisons were performed using the independent 2-sample *t* test or the Mann-Whitney *U* test for continuous variables, and the chi-square or Fisher exact test for categorical variables, as appropriate.

For intergroup comparison, the incidence of the composite outcome was compared between the AI-ECG high- and low-risk groups within the Revised Cardiac Risk Index (RCRI) and European Society of Cardiology (ESC) surgical risk strata. The incidence rates with 95% CIs, relative risks (RRs), and risk differences were derived from contingency 2×2 tables comparing AI-ECG risk groups and the presence or absence of the composite outcome. Within each AI-ECG stratum, the outcome incidence across the three conventional testing groups (no advanced cardiovascular imaging, negative test, and positive test) was compared using the chi-square or Fisher exact tests; pairwise Fisher exact tests and Bonferroni correction were applied when appropriate. For comparisons across multiple strata, overall differences were evaluated using 1-way ANOVA or the Kruskal-Wallis test for continuous variables, and the chi-square or Fisher exact test for categorical variables. To assess the incremental prognostic value of incorporating the AI-ECG into RCRI, we calculated the change in the area under the receiver operating characteristic curve, the continuous net reclassification improvement (NRI), and the integrated discrimination improvement (IDI). We also constructed a generalized estimating equation (GEE) model setting the patient identifier as the cluster variable to address potential within-patient correlation.

To assess the robustness of our findings, we conducted prespecified sensitivity analyses across surgical subgroups: major surgery under general anesthesia, elective surgery, and emergency surgery. Furthermore, to address potential within-patient correlation arising from multiple surgeries per patient, we performed a patient-level sensitivity analysis restricted to each patient’s first index surgery. In addition, to further address potential confounding by indication, we performed propensity score matching (PSM). Surgeries with and without advanced cardiovascular testing were matched in a 1:1 ratio using nearest-neighbor matching (caliper=0.2). The matching model included critical confounders: emergency surgery status, age, sex, type of anesthesia, ESC surgical risk category, RCRI score, American Society of Anesthesiologists class, and comorbidities.

Following STROBE guidelines, missing values were identified only for smoking status (7795/46,135, 16.9%) and baseline serum creatinine (n=14). As these variables were solely used for baseline descriptive statistics, no missing data imputation was performed. Missing smoking status was presented as a separate “unknown” category. Statistical significance was defined using a 2-sided *P* value <.05. All analyses were performed using R (version 4.4.1; R Foundation).

## Results

### Baseline Characteristics

Baseline clinical and surgery-specific characteristics of this study’s population are summarized in Table 1. The mean age was 56.6 (SD 16.1) years, and males accounted for 44.6% (20,561/46,135) of the cases. Emergency procedures comprised 6.1% (2806/46,135) of all surgeries, and 72.4% (33,386/46,135) of them were performed under general anesthesia. According to the ESC surgical risk categorization and the RCRI stratification, high-risk surgery was identified in 10.7% (4918/46,135) and 4.2% (1929/46,135) of cases, respectively.

Overall, 3536 (7.7%) ECG–surgery pairs had at least one QCG value that exceeded the predefined cutoffs and were thus classified as high-risk by AI-ECG. Compared to the low-risk group, high-risk cases involved older individuals, had a higher proportion of males, presented with a greater burden of comorbidities, underwent a higher proportion of emergency procedures, and were more frequently categorized as undergoing high-risk surgeries, according to both the ESC and RCRI (Table 1) risk categorizations.

The distribution of the QCG scores across the six study groups is shown in Figure S2 in Multimedia Appendix 1, with significantly higher scores observed in the AI-ECG–stratified high-risk group. Across the three strata of preoperative cardiovascular test results, cases in the positive-test group involved the oldest individuals, had the highest comorbidity burden, underwent high-risk surgery, and had the highest QCG scores. Cases in the negative-test group showed intermediate characteristics, whereas those in the no advanced cardiovascular imaging group involved the youngest individuals, with the fewest comorbidities and the lowest QCG scores (Table S2 in Multimedia Appendix 1).

**Table 1:** Baseline characteristics and comparison between AI-ECG^a^ risk groups.

Variable			Overall (N=46,135)	AI-ECG low-risk (n=42,599)	AI-ECG high-risk (n=3536)	*P* value	
Demographics and comorbidities							
	Age (years), mean (SD)		56.6 (16.1)	55.7 (16.0)	67.4 (14.0)	<.001	
	Male sex (%)		20,561 (44.6)	18,283 (42.9)	2278 (64.4)	<.001	
	DM^b^ (%)		5904 (12.8)	4901 (11.5)	1003 (28.4)	<.001	
		DM treated with insulin (%)	632 (1.4)	444 (1.0)	188 (5.3)	<.001	
	Hypertension (%)		10,759 (23.3)	9297 (21.8)	1462 (41.3)	<.001	
	Previous heart failure (%)		410 (0.9)	139 (0.3)	271 (7.7)	<.001	
	Previous ischemic heart disease (%)		1772 (3.8)	1115 (2.6)	657 (18.6)	<.001	
	Previous cerebrovascular accident (%)		1525 (3.3)	1213 (2.8)	312 (8.8)	<.001	
	Smoking (%)						<.001
		Nonsmoker	28,436 (61.6)	26,343 (61.8)	2093 (59.2)		
		Smoker	9904 (21.5)	8769 (20.6)	1135 (32.1)		
		Unknown	7795 (16.9)	7487 (17.6)	308 (8.7)		
	Creatinine, median (IQR)		0.74 (0.62-0.91)	0.73 (0.61-0.89)	0.90 (0.71-1.26)	<.001	
	Creatinine ≥2.0 mg/dl (%)		1414 (3.1)	850 (2.0)	564 (16.0)	<.001	
Surgery-related							
	Emergency surgery (%)		2806 (6.1)	2337 (5.5)	469 (13.3)	<.001	
	Anesthesia (%)						<.001
		General anesthesia	33,386 (72.4)	30,946 (72.6)	2440 (69.0)		
		Spinal or epidural anesthesia	4272 (9.3)	3964 (9.3)	308 (8.7)		
		Monitored anesthesia care	8477 (18.4)	7689 (18.0)	788 (22.3)		
	ESC^c^ surgical risk category (%)						<.001
		Low	24,825 (53.8)	23,280 (54.6)	1545 (43.7)		
		Intermediate	16,392 (35.5)	15,161 (35.6)	1231 (34.8)		
		High	4918 (10.7)	4158 (9.8)	760 (21.5)		
	RCRI^d^ (%)						<.001
		0	28,738 (62.3)	27,427 (64.4)	1311 (37.1)		
		1	15,468 (33.5)	14,009 (32.9)	1459 (41.3)		
		2	1562 (3.4)	1011 (2.4)	551 (15.6)		
		3	308 (0.7)	137 (0.3)	171 (4.8)		
		4	50 (0.1)	15 (0.0)	35 (1.0)		
		5	9 (0.0)	0 (0.0)	9 (0.3)		
	ASA^e^ classification (%)						<.001
		1	13,814 (29.9)	13,623 (32.0)	191 (5.4)		
		2	24,130 (52.3)	22,955 (53.9)	1175 (33.2)		
		3	7332 (15.9)	5591 (13.1)	1741 (49.2)		
		4	765 (1.7)	377 (0.9)	388 (11.0)		
		5	94 (0.2)	53 (0.1)	41 (1.2)		
QCG^f^ scores, median (IQR)							
	ACS^g^		1.37 (0.30-4.26)	1.20 (0.27-3.62)	12.68 (2.84-25.99)	<.001	
	STEMI^h^		0.01 (0.00-0.07)	0.01 (0.00-0.05)	0.41 (0.06-1.95)	<.001	
	Myocardial injury		1.71 (0.50-4.58)	1.48 (0.44-3.79)	16.80 (6.49-26.29)	<.001	
	Pulmonary edema		0.63 (0.20-2.18)	0.55 (0.18-1.72)	15.22 (2.81-26.98)	<.001	
	Pericardial effusion		0.03 (0.00-0.23)	0.02 (0.00-0.16)	1.34 (0.10-7.40)	<.001	
	Left ventricular dysfunction		0.08 (0.02-0.34)	0.07 (0.02-0.24)	4.16 (0.70-19.20)	<.001	
	Right ventricular dysfunction		0.13 (0.04-0.57)	0.11 (0.03-0.41)	5.02 (0.75-17.61)	<.001	
	Pulmonary hypertension		0.15 (0.03-0.76)	0.12 (0.03-0.56)	5.60 (0.64-17.59)	<.001	
Preoperative cardiovascular test							
	Any preoperative CV^i^ test performed (%)		5458 (11.8)	4251 (10.0)	1207 (34.1)	<.001	
		Positive any preoperative CV test (%)	892 (16.3)	383 (9.0)	509 (42.2)	<.001	
		Echocardiography performed (%)	4808 (10.4)	3842 (9.0)	966 (27.3)	<.001	
		Positive echocardiography results (%)	574 (11.9)	243 (6.3)	331 (34.3)	<.001	
		Moderate or severe valvular heart disease (%)	235 (4.9)	114 (3.0)	121 (12.6)	<.001	
		Regional wall motion abnormality (%)	374 (7.8)	130 (3.4)	244 (25.5)	<.001	
		LVEF^j^ <50% (%)	225 (4.7)	54 (1.4)	171 (17.8)	<.001	
		LVEF (%), median (IQR)	62.7 (58.9-66.3)	63.2 (59.7-66.7)	59.9 (54.1-64.8)	<.001	
	CAG^k^ performed (%)		470 (1.0)	183 (0.4)	287 (8.1)	<.001	
		Positive CAG results (%)	273 (58.1)	76 (41.5)	197 (68.6)	<.001	
	Coronary CT^l^ angiography performed (%)		579 (1.3)	411 (1.0)	168 (4.8)	<.001	
		Positive coronary CT angiography results (%)	165 (29.0)	99 (24.4)	66 (40.5)	<.001	
	SPECT^m^ performed (%)		245 (0.5)	150 (0.4)	95 (2.7)	<.001	
		Positive SPECT results (%)	7 (2.9)	3 (2.0)	4 (4.2)	.54	

^a^AI-ECG: artificial intelligence–enabled electrocardiography.

^b^DM: diabetes mellitus.

^c^ESC: European Society of Cardiology.

^d^RCRI: Revised Cardiac Risk Index.

^e^ASA: American Society of Anesthesiologists.

^f^QCG: quantitative score for electrocardiography.

^g^ACS: acute coronary syndrome.

^h^STEMI: ST-segment elevation myocardial infarction.

^i^CV: cardiovascular.

^j^LVEF: left ventricular ejection fraction.

^k^CAG: coronary angiography.

^l^CT: computed tomography.

^m^SPECT: single-photon emission computed tomography.

### Postoperative Outcomes

In total, 180 (0.4%) cases experienced the postoperative composite outcome. Overall, the event rates were 2.9% and 0.2% in the high-risk and low-risk AI-ECG groups, respectively. When stratified by surgical urgency, composite outcomes occurred in 2.8% (78/2806) and 0.2% (102/43,329) of emergency and elective surgeries, respectively. Among the emergency surgery cases, the risks of the composite end point were 8.5% and 1.6% in the AI-ECG–stratified high-risk and low-risk groups, respectively.

The incidence of the composite outcome was consistently higher in AI-ECG–graded high-risk cases across all ESC surgical risk and RCRI strata. In the ESC surgical risk- and RCRI-categorized low-risk procedures, the RRs of the composite outcome occurrence were higher in the low-risk AI-ECG group than in the high-risk AI-ECG group (RR 58.1, 95% CI 25.4-133.3) and 14.3 (10.1–20.1) for ESC surgical risk and RCRI, respectively, while the procedures classified as high-risk by the established risk stratification tools showed larger risk differences between the low-risk and high-risk AI-ECG groups (Table 2). Figure S3 in Multimedia Appendix 1 demonstrated that AI-ECG improved patient reclassification compared to conventional risk stratification systems. Incorporating the AI-ECG into a baseline model including age and RCRI provided significant incremental prognostic value, as demonstrated by improved area under the receiver operating characteristic curve, NRI, and IDI (Figure S4 in Multimedia Appendix 1). Notably, while the overall classification improvement was significant (NRI 0.675, *P*<.001), it was primarily driven by the accurate down-classification of nonevents (NRI for nonevents 0.530, *P*<.001), whereas the reclassification for events did not reach statistical significance (NRI for events 0.144, *P*=.05). Furthermore, in the additional analysis accounting for within-patient correlation using GEE, the high-risk designation by AI-ECG remained a significant predictor of the primary composite outcome after adjustment for age and RCRI (adjusted GEE odds ratio 8.26, 95% CI 5.93-11.50, *P*<.001).

**Table 2:** The incidence, relative risk, and relative difference of the composite outcome across ESC^a^ surgical risk category and RCRI^b^ strata by AI-ECG^c^ risk stratification.

Risk group		Incidence of composite outcomes			
		AI-ECG–stratified, low-risk (%)	AI-ECG–stratified, high-risk (%)	RR^d^ (95% CI)	RD^e^ %*P* (95% CI)
Surgical emergency					
	Elective	0.10 (0.07-0.14)	1.99 (1.52-2.55)	19.53 (13.17-28.97)	1.89 (1.39-2.38)
	Emergency	1.63 (1.15-2.23)	8.53 (6.16-11.43)	5.25 (3.40-8.09)	6.90 (4.32-9.48)
ESC surgical risk					
	Low	0.03 (0.01-0.06)	1.75 (1.15-2.53)	58.12 (25.35-133.25)	1.72 (1.06-2.37)
	Intermediate	0.31 (0.23-0.41)	3.01 (2.12-4.12)	9.70 (6.33-14.86)	2.70 (1.74-3.65)
	High	0.60 (0.39-0.89)	4.87 (3.45-6.65)	8.10 (4.90-13.37)	4.27 (2.72-5.82)
RCRI					
	0-1	0.16 (0.12-0.20)	2.27 (1.75-2.90)	14.28 (10.13-20.12)	2.12 (1.56-2.67)
	2+	1.12 (0.60-1.90)	4.96 (3.53-6.75)	4.44 (2.38-8.28)	3.84 (2.19-5.50)
Total		0.19 (0.15-0.23)	2.86 (2.33-3.46)	15.40 (11.50-20.63)	2.67 (2.12-3.22)

^a^ESC: European Society of Cardiology.

^b^RCRI: Revised Cardiac Risk Index.

^c^AI-ECG: artificial intelligence–enabled electrocardiography.

^d^RR: relative risk.

^e^RD: relative difference.

Evaluated as a stand-alone tool, the AI-ECG yielded a negative predictive value of 99.8% and a positive predictive value (PPV) of 2.9%. Considering the extremely low baseline event rate (0.4%), this PPV reflects a 7.4-fold increased RR, albeit representing a modest absolute predictive value. Furthermore, these risk stratification capabilities were consistently maintained when evaluating the exploratory composite outcome that included prolonged mechanical ventilation (Table S3 in Multimedia Appendix 1).

**Figure 2:**
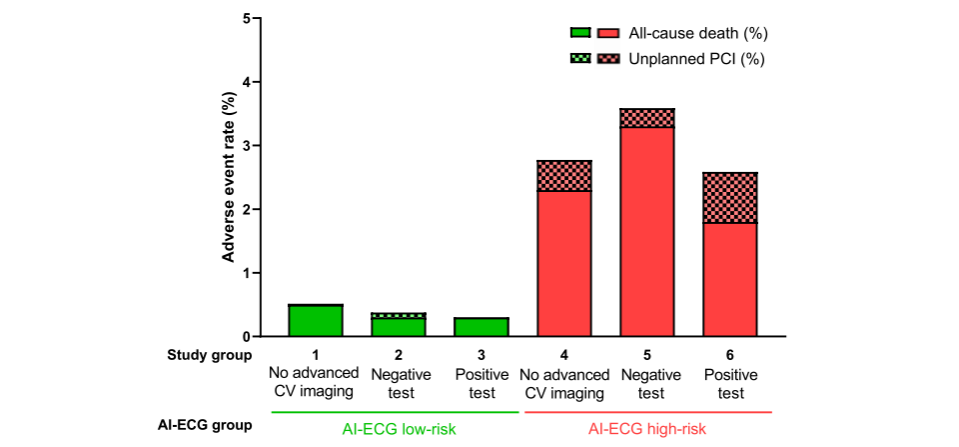
Predefined outcome rates across the six study groups assigned by the AI-ECG (low-risk green; high-risk red) and the preoperative CV testing. Death represents all-cause mortality; cardiac death was not adjudicated separately in this study. AI-ECG: artificial intelligence–enabled electrocardiography; CV: cardiovascular; PCI: percutaneous coronary intervention.

Comparisons of predefined outcomes across the six study groups, defined by AI-ECG and preoperative cardiovascular test results, are summarized in Figure 2. The composite outcomes differed significantly between the low-risk and high-risk groups on AI-ECG (*P*<.001 for each comparison within the no advanced cardiovascular imaging, negative-test, and positive-test groups). The low-risk AI-ECG group showed consistently low composite outcome rates compared to the high-risk AI-ECG group, irrespective of advanced cardiovascular imaging. Within the AI-ECG low-risk group, although a nominal statistical significance was observed among the subgroups (0.2%, 0.4%, and 0.3% for the no advanced cardiovascular imaging, negative-test, and positive-test groups, respectively; *P*=.025), the absolute event rates remained uniformly low. Within the high-risk AI-ECG group, there was no significant intragroup difference in the outcome incidences (2.8%, 3.4%, and 2.6%, respectively; *P*=.572, Table 3).

**Table 3:** Predefined outcome rates across the six study groups assigned by the artificial intelligence–enabled electrocardiography and the preoperative cardiovascular testing.

Study group		Group 1 (n=38,348)	Group 2 (n=3868)	Group 3 (n=383)	Group 4 (n=2329)	Group 5 (n=698)	Group 6 (n=509)	*P* value
Composite outcome, n (%)		64 (0.2)	14 (0.4)	1 (0.3)	64 (2.8)	24 (3.4)	13 (2.6)	<.001
	All-cause death, n (%)	58 (0.2)	11 (0.3)	1 (0.3)	54 (2.3)	23 (3.3)	9 (1.8)	<.001
	Unplanned percutaneous coronary intervention, n (%)	6 (0.02)	3 (0.1)	0 (0.0)	11 (0.5)	2 (0.3)	4 (0.8)	<.001

### Preoperative Cardiovascular Test and Reclassification Using AI-ECG

Preoperative cardiovascular testing was performed in 11.8% (5458/46,135) of the cases, with only 16.3% (892/46,135) showing positive findings. Of the cases that involved preoperative cardiac testing, 88.1% (4808/5458) included echocardiography, making it the most commonly used modality. Among the cases in which preoperative cardiovascular testing was performed (groups 2, 3, 5, and 6), the negative-test groups (groups 2 and 5) were less likely to involve additional cardiovascular tests beyond echocardiography than the positive-test groups (groups 3 and 6). The types and results of preoperative cardiovascular tests are presented in Table S4 in Multimedia Appendix 1.

**Figure 3:**
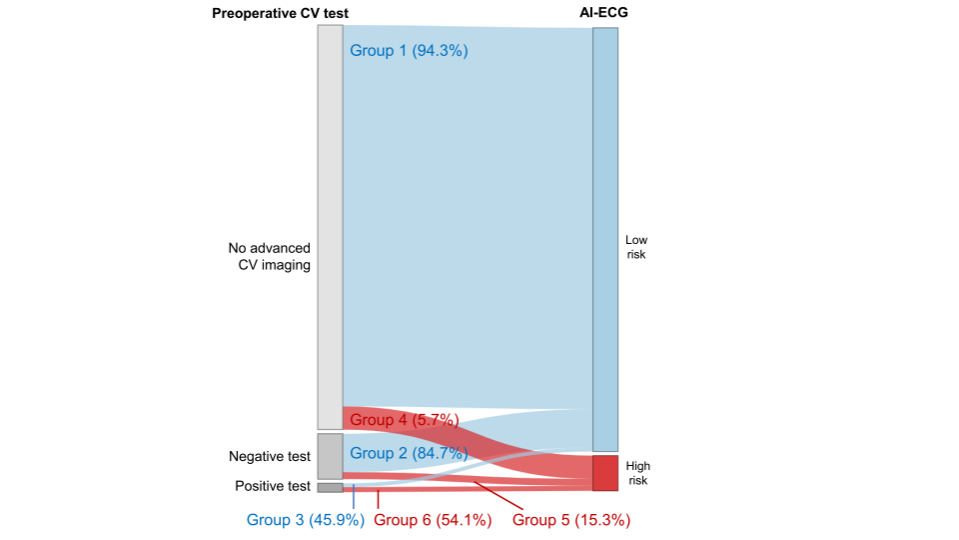
The proportion of AI-ECG risk according to the preoperative CV test. A substantial proportion of ECG-surgery pairs in the no advanced CV imaging group (2329/40,677, 5.7%) were classified as high-risk by AI-ECG (red), whereas the majority of cases in the negative-test group (3868/4566, 84.7%) were classified as low-risk by AI-ECG (light blue). AI-ECG: artificial intelligence–enabled electrocardiography; CV: cardiovascular; ECG: electrocardiography.

When stratified by preoperative cardiovascular testing, all outcomes except for prolonged mechanical ventilation in the positive-test group showed significant differences according to the AI-ECG risk level. As illustrated in Figure 3, a substantial proportion of cases in the no advanced cardiovascular imaging group (2329/40,677, 5.7%) were classified as high-risk by AI-ECG, whereas most cases in the negative-test group (3868/4566, 84.7%) were classified as low-risk.

### Assessment for Individual Study Groups

The characteristics of each study group are summarized in Table 4. Groups 1 and 6 demonstrated appropriate application of the AI-ECG–derived risk classification: group 1 included low-risk cases without further testing, and group 6 included high-risk cases in which testing yielded abnormal findings. Accordingly, the subsequent evaluation focuses on groups 2-5.

**Figure 4:**
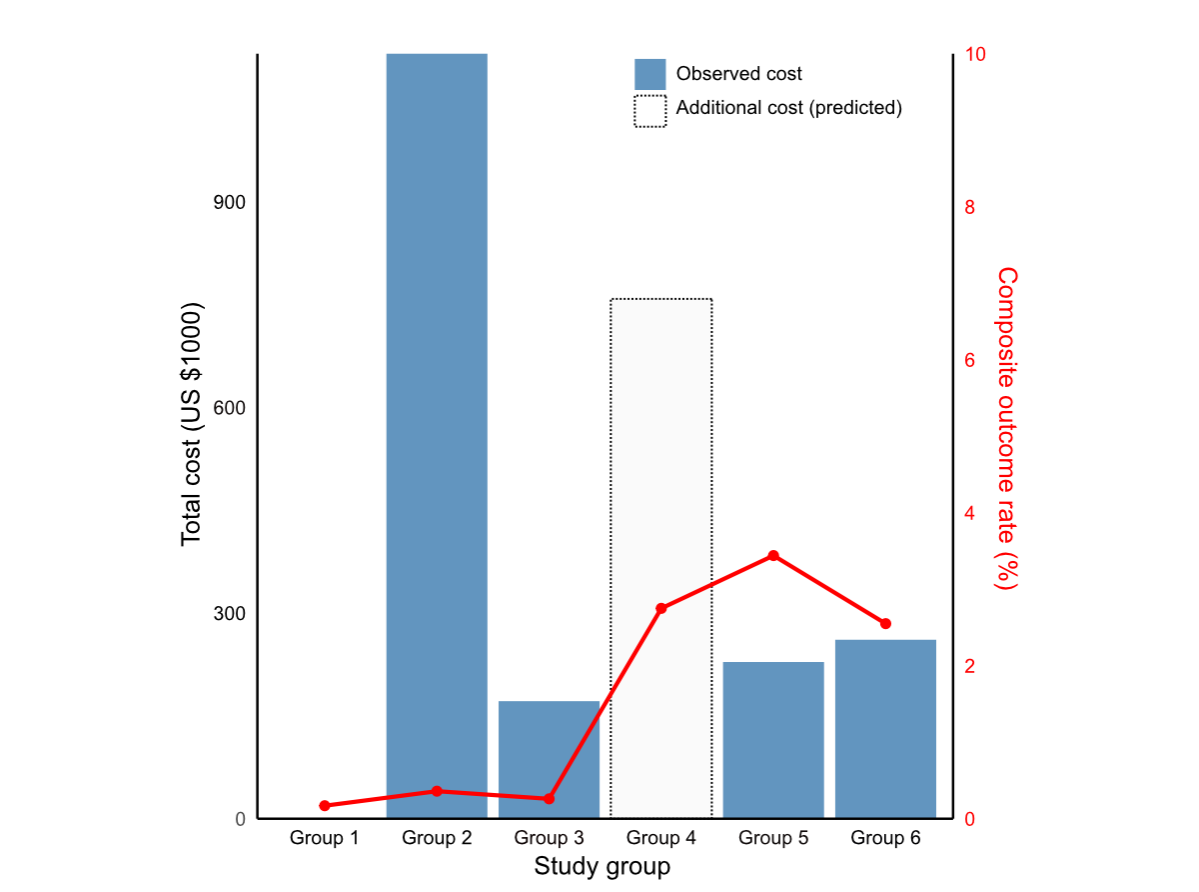
Total cost and composite outcome rate by six study groups. The bar graphs represent the total cost observed (filled with blue), and the predicted additional cost by assuming that cases in group 4 undergo advanced cardiovascular imaging according to the proportion of individual tests (the part shown in dotted line), respectively (US $1000). The red line graph represents the composite outcome rate (%).

Group 4 comprised cases classified as high-risk by AI-ECG that proceeded to surgery without advanced cardiovascular imaging. This group had the highest frequency of emergency procedures (n=389); otherwise, most cases were categorized as low-risk surgery by the RCRI (n=1991) or ESC surgical risk (n=1147; Figure S5 in Multimedia Appendix 1). Among the remaining cases (n=137) after excluding these two reasons, 105 procedures involved preoperative cardiovascular testing at another hospital. Thus, the number of “true” missing cases by clinicians was 32, in which no further diagnostic tests or cardiology consultations were undertaken despite high-risk AI-ECG findings. Among these 32 cases, two deaths occurred (mortality rate: 6.3%). Although preoperative advanced cardiovascular testing was not performed in group 4, downstream costs were estimated by applying the mean frequency of individual tests observed in cases involving at least one of the advanced cardiovascular tests (groups 2, 3, 5, and 6; Figure 4).

Group 5 included cases classified as high-risk by AI-ECG, but with negative preoperative cardiovascular tests. This group consisted of a heterogeneous set of conditions: (1) abnormalities detected by preoperative cardiovascular testing that were not classified as positive results (eg, atrial fibrillation, myocardial disease, and severe left ventricular hypertrophy), (2) a history of open-heart surgery but with preserved cardiac function, or (3) stable coronary stenosis or prior PCI without interval change. Group 5 had the highest composite end point rate (3.4%).

Group 2 included ECG–surgery pairs classified as low-risk by AI-ECG that involved preoperative advanced cardiovascular imaging with negative results. The most frequently performed test modality in this group was echocardiography (3553/3868, 91.9%), which resulted in the highest cost of preoperative cardiovascular tests among the six study groups (Figure 4). The proportion of the cost spent in group 2 was 62.8% (USD $1,115,966,000/$1,777,059,000) of the total spending on preoperative cardiovascular evaluations.

Group 3 represents cases with abnormal findings on advanced cardiovascular testing despite a low-risk classification by AI-ECG. In this group, composite outcomes occurred in 1 patient (0.3%, death).

**Table 4:** Characteristics of individual study groups.

				Group 1 (n=38,348)		Group 2 (n=3868)	Group 3 (n=383)		Group 4 (n=2329)		Group 5 (n=698)		Group 6 (n=509)		*P* value	
Demographics and comorbidities																
	Age (years), mean (SD)		54.2 (15.7)		68.8 (11.7)		72.0 (10.4)	65.3 (14.3)		71.5 (12.9)		71.5 (11.8)		<.001		
	Male sex (%)		16,207 (42.3)		1820 (47.1)		256 (66.8)	1538 (66.0)		399 (57.2)		341 (67.0)		<.001		
	DM^a^ (%)		4001 (10.4)		796 (20.6)		104 (27.2)	629 (27.0)		172 (24.6)		202 (39.7)		<.001		
		DM treated with insulin (%)	315 (0.8)		112 (2.9)		17 (4.4)	96 (4.1)		47 (6.7)		45 (8.8)		<.001		
	Hypertension (%)		7557 (19.7)		1553 (40.1)		187 (48.8)	935 (40.1)		276 (39.5)		251 (49.3)		<.001		
	Previous heart failure (%)		44 (0.1)		32 (0.8)		63 (16.4)	66 (2.8)		17 (2.4)		188 (36.9)		<.001		
	Previous ischemic heart disease (%)		702 (1.8)		263 (6.8)		150 (39.2)	288 (12.4)		82 (11.7)		287 (56.4)		<.001		
	Previous cerebrovascular accident (%)		965 (2.5)		203 (5.2)		45 (11.7)	199 (8.5)		57 (8.2)		56 (11.0)		<.001		
	Smoking (%)															<.001
		Nonsmoker	23,402 (61.0)		2735 (70.7)		206 (53.8)	1328 (57.0)		482 (69.1)		283 (55.6)				
		Smoker	7577 (19.8)		1034 (26.7)		158 (41.3)	722 (31.0)		200 (28.7)		213 (41.8)				
		Unknown	7369 (19.2)		99 (2.6)		19 (5.0)	279 (12.0)		16 (2.3)		13 (2.6)				
	Creatinine, median (IQR)		0.72 (0.61-0.88)		0.78 (0.64-0.95)		0.88 (0.73-1.10)	0.89 (0.70-1.20)		0.88 (0.68-1.26)		1.01 (0.79-1.65)		<.001		
		Creatinine ≥2.0 mg/dl (%)	670 (1.7)		147 (3.8)		33 (8.6)	358 (15.4)		96 (13.8)		110 (21.6)		<.001		
Surgery-related																
	Emergency surgery (%)		2230 (5.8)		88 (2.3)		19 (5.0)	389 (16.7)		44 (6.3)		36 (7.1)		<.001		
	Anesthesia (%)															<.001
		General anesthesia	27,687 (72.2)		2965 (76.7)		294 (76.8)	1576 (67.7)		525 (75.2)		339 (66.6)				
		Spinal/epidural anesthesia	3224 (8.4)		698 (18.0)		42 (11.0)	163 (7.0)		94 (13.5)		51 (10.0)				
		Monitored anesthesia care	7437 (19.4)		205 (5.3)		47 (12.3)	590 (25.3)		79 (11.3)		119 (23.4)				
	ESC^b^ surgical risk category (%)															<.001
		Low	22,098 (57.6)		1080 (27.9)		102 (26.6)	1147 (49.2)		206 (29.5)		192 (37.7)				
		Intermediate	12,821 (33.4)		2191 (56.6)		149 (38.9)	740 (31.8)		322 (46.1)		169 (33.2)				
		High	3429 (8.9)		597 (15.4)		132 (34.5)	442 (19.0)		170 (24.4)		148 (29.1)				
	RCRI^c^ (%)															<.001
		0	25,503 (66.5)		1855 (48.0)		69 (18.0)	1009 (43.3)		256 (36.7)		46 (9.0)				
		1	12,145 (31.7)		1693 (43.8)		171 (44.6)	982 (42.2)		312 (44.7)		165 (32.4)				
		2	624 (1.6)		280 (7.2)		107 (27.9)	261 (11.2)		104 (14.9)		186 (36.5)				
		3	69 (0.2)		34 (0.9)		34 (8.9)	54 (2.3)		25 (3.6)		92 (18.1)				
		4	7 (0.0)		6 (0.2)		2 (0.5)	18 (0.8)		1 (0.1)		16 (3.1)				
		5	0 (0.0)		0 (0.0)		0 (0.0)	5 (0.2)		0 (0.0)		4 (0.8)				
	ASA^d^ classification (%)															<.001
		1	13,389 (34.9)		233 (6.0)		1 (0.3)	179 (7.7)		12 (1.7)		0 (0.0)				
		2	20,544 (53.6)		2319 (60.0)		92 (24.0)	907 (38.9)		226 (32.4)		42 (8.3)				
		3	4142 (10.8)		1218 (31.5)		231 (60.3)	1034 (44.4)		377 (54.0)		330 (64.8)				
		4	226 (0.6)		93 (2.4)		58 (15.1)	175 (7.5)		77 (11.0)		136 (26.7)				
		5	47 (0.1)		5 (0.1)		1 (0.3)	34 (1.5)		6 (0.9)		1 (0.2)				
QCG^e^ scores, median (IQR)																
	ACS^f^		1.04 (0.22-3.28)		2.80 (1.18-6.22)		5.70 (3.00-10.76)	13.05 (2.30-25.76)		8.30 (2.77-23.89)		16.95 (5.60-32.72)		<.001		
	STEMI^g^		0.01 (0.00-0.04)		0.03 (0.01-0.14)		0.15 (0.03-0.60)	0.34 (0.05-1.51)		0.40 (0.06-1.65)		1.40 (0.23-6.80)		<.001		
	Myocardial injury		1.32 (0.39-3.46)		3.16 (1.47-6.10)		5.91 (3.36-9.60)	16.82 (5.80-25.89)		14.22 (6.28-23.58)		19.50 (10.23-33.50)		<.001		
	Pulmonary edema		0.49 (0.16-1.50)		1.51 (0.53-4.02)		4.00 (1.29-8.99)	12.02 (1.72-25.26)		18.05 (4.34-26.97)		21.60 (9.10-36.38)		<.001		
	Pericardial effusion		0.02 (0.00-0.14)		0.06 (0.01-0.45)		0.27 (0.04-1.58)	0.89 (0.05-6.10)		2.48 (0.22-9.80)		3.10 (0.70-9.10)		<.001		
	Left ventricular dysfunction		0.06 (0.02-0.21)		0.15 (0.04-0.61)		0.95 (0.30-3.75)	3.07 (0.47-16.05)		3.50 (0.72-13.14)		21.00 (5.41-54.70)		<.001		
	Right ventricular dysfunction		0.10 (0.03-0.36)		0.26 (0.08-1.10)		1.10 (0.32-3.65)	3.80 (0.48-15.12)		5.62 (1.11-16.47)		10.90 (3.90-26.60)		<.001		
	Pulmonary hypertension		0.11 (0.03-0.48)		0.40 (0.09-1.72)		1.45 (0.30-5.27)	4.05 (0.37-15.08)		7.90 (1.43-19.79)		10.77 (3.40-25.20)		<.001		

^a^DM: diabetes mellitus.

^b^ESC: European Society of Cardiology.

^c^RCRI: Revised Cardiac Risk Index.

^d^ASA: American Society of Anesthesiologists.

^e^QCG: quantitative score for electrocardiography.

^f^ACS: acute coronary syndrome.

^g^STEMI: ST-segment elevation myocardial infarction.

### Sensitivity Analysis

When analyses were restricted to major surgery under general anesthesia, elective surgeries, and emergency surgeries, the association between AI-ECG risk stratification and postoperative outcomes remained consistent. In group-wise analyses, groups classified as high-risk by AI-ECG (groups 4, 5, and 6) consistently exhibited higher adverse event rates than those classified as low-risk (groups 1, 2, and 3) across all surgical subgroups (Table S5 in Multimedia Appendix 1). Similarly, in the patient-level sensitivity analysis restricted to each patient’s first surgery (n=41,218), the trend in event rates and RRs remained consistent with those of the primary analysis (Table S6 in Multimedia Appendix 1).

To further address potential confounding by indication regarding preoperative evaluation, we additionally performed a propensity score-matched analysis. In the balanced cohort of 5122 well-matched pairs (n=10,244), the overall trends in composite outcomes remained highly consistent with our primary unmatched analysis, confirming the robustness of the AI-ECG risk stratification (Tables S7 and S8 in Multimedia Appendix 1).

## Discussion

### Principal Findings

In this study, we demonstrated the potential utility of multitask AI-enhanced ECG in reducing the tests with limited incremental diagnostic value and the associated costs in preoperative risk assessment. Among noncardiac surgeries, >90% were classified as low-risk by AI-ECG, with a high negative predictive value of the AI-ECG low-risk group designation for the primary outcome, supporting the utility of AI-ECG as a rule-out screening tool in the preoperative setting. Notably, in those who underwent preoperative cardiovascular testing despite a low-risk AI-ECG classification, 91% had negative results; however, this negative-test group incurred more than half of the overall expenditure for preoperative cardiovascular testing in all surgeries. When the AI-ECG risk classification was integrated with traditional risk-stratification tools, such as the ESC surgical risk category and RCRI, cases identified as low-risk had an extremely low incidence of postoperative composite outcomes. This potentially enables the conservation of medical resources by minimizing low-yield advanced cardiovascular evaluation before noncardiac surgery.

The indiscriminate use of preoperative cardiovascular tests has raised several important concerns. Overuse not only increases health care costs and delays surgery but may also postpone tests intended for diagnosing other urgent conditions. Furthermore, the prevalence of serious cardiac diseases that require a change in surgical plan is low in elective surgery candidates, unless they have cardiac symptoms or signs, resulting in a low yield of meaningful results [23]. Nevertheless, preoperative cardiovascular tests, especially echocardiography, are still being performed even before low-risk surgery in a substantial number of cases. In a retrospective study of low-risk surgery, 39.8% of cases underwent echocardiography, among which only 5% showed abnormal results [24]. Moreover, among the diverse findings that could be obtained from echocardiography, the guidelines for perioperative cardiac management require only left and right ventricular function as preoperative cardiovascular diagnostic testing. Consequently, echocardiography is not recommended routinely in asymptomatic and clinically stable patients before noncardiac surgery owing to the lack of proven benefit [5]. In the meantime, no single cardiovascular test can fully assess perioperative risk. For example, normal echocardiography findings cannot reliably exclude ischemic heart disease without complementary stress tests. Importantly, postoperative cardiac complications are heterogeneous, ranging from ACS requiring unplanned PCI to heart failure resulting in prolonged intensive care unit stay and even mortality, which cannot be predicted by a single diagnostic modality. Taken together, these limitations highlight the unmet need for a novel and more comprehensive risk-stratification tool to identify high-risk surgical candidates who require further preoperative cardiovascular evaluation. Such a tool would enable the more appropriate use of limited health care resources, reduce overusage of testing, and ultimately improve perioperative outcomes.

In this study, we introduced a multitask AI-ECG, originally developed to detect patients at risk of a wide spectrum of cardiac conditions, including ST-segment elevation myocardial infarction, ACS, left and right ventricular dysfunction, pulmonary edema, pulmonary hypertension, and pericardial effusion, as a novel tool to identify surgical candidates at a low-risk of composite postoperative outcomes. When all eight QCG scores were below the optimal threshold, we designated this surgery as low-risk. To evaluate the clinical utility of this approach, we categorized all surgery-ECG pairs into three groups according to the results of the preoperative cardiovascular testing: no advanced cardiovascular imaging, negative-test, and positive-test. Each group was further stratified according to the AI-ECG risk classification (low-risk vs high-risk), resulting in six subgroups. Assuming that the decision to perform preoperative cardiovascular testing strictly followed the AI-ECG risk stratification (ie, tests performed only in high-risk cases), the number of surgeries in which cardiovascular tests were performed (groups 2, 3, 5, and 6; n=5458) exceeded the number of surgeries in which testing would have been required (groups 4, 5, and 6; n=3536). In a hypothetical scenario assuming strict adherence to the AI-ECG guidance, this discrepancy suggests a potential reduction of up to 35.2% in the usage of low-yield preoperative cardiovascular testing (Figure 1). Given that multiple cardiovascular tests may be required in some patients before noncardiac surgery, preoperative evaluation using multitask AI-ECG could be more practical by providing multiple disease-specific risk scores simultaneously from a single ECG image. In contrast to other machine learning algorithms that rely on numerous clinical variables from electronic health care records to predict postoperative adverse events [25-27], our platform enables simple and efficient on-site risk stratification using only a single ECG.

### Group-Wise Analysis

In group-wise analysis, group 2 emerged as the most notable subgroup. Despite being classified as low-risk by AI-ECG and demonstrating a low prevalence of composite outcomes, this group (n=3868) accounted for more than half of the total patient expenses. On the other hand, group 4 (n=2329), characterized by a high AI-ECG risk, did not undergo testing despite their higher event rate. Taken together, approximately 40% (1539/3868) of the cardiovascular tests might be considered as overused testing, and the composite outcome incidence was markedly higher in group 4 than in group 2. As the symptoms and signs that led to advanced cardiovascular imaging were not accounted for in our assumption, all tests in group 2 should not be treated as unnecessary. However, this finding underscores that AI-ECG not only potentially decreases testing but also provides more appropriate risk stratification.

Group 3 represents another potentially “missed” population under an AI-ECG–guided strategy. Importantly, a low-risk AI-ECG classification in group 3 does not imply the absence of cardiovascular disease but rather reflects lower physiological vulnerability at the time of surgery. Additionally, this must be interpreted with caution due to the treatment paradox and verification bias. The favorable outcomes observed in group 3 may be a direct result of intense medical optimization, altered anesthetic management, and heightened postoperative monitoring initiated by the abnormal test findings, rather than confirming that the advanced cardiovascular testing was unnecessary.

Group 6—cases classified as high-risk by AI-ECG with positive preoperative cardiovascular testing—demonstrated relatively lower event rates compared to groups 4 and 5, despite carrying the highest burden of structural cardiovascular disease. As with group 3, this may be partly explained by the fact that a subset of group 6 cases involved intensified perioperative management, such as coronary revascularization before surgery. Figure S6 in Multimedia Appendix 1 illustrates a representative case where AI-ECG risk scores significantly decreased after the revascularization procedure. This temporal change supports the interpretation that AI-ECG reflects dynamic physiological cardiac state, capturing risk modification after therapeutic intervention rather than solely reflecting static anatomical disease burden. Furthermore, the paradoxically higher event rate in group 5 (negative cardiovascular testing) than in group 6 suggests the limited ability of conventional imaging to completely rule out the residual cardiovascular risk.

Notably, when VHD was analyzed in detail, severe VHD was more frequently observed in group 6 than in group 3, and markers of advanced physiological burden, including reduced left ventricular ejection fraction and multiple severe valvular lesions, were largely confined to group 6 (Table S4 in Multimedia Appendix 1). These findings suggest that even among cases with structural heart disease, AI-ECG preferentially classified those with greater physiological burden into the high-risk group. Nevertheless, we acknowledge that structural valvular abnormalities that do not manifest prominent electrical changes bear an inherent limitation of ECG-based models. Accordingly, a low-risk AI-ECG result should not be interpreted as a substitute for echocardiography in cases with clinical findings suggestive of valvular disease. We suggest a stepwise pipeline where a standard physical examination (especially auscultation) is followed by the AI-ECG to triage the need for advanced cardiovascular tests, rather than using the AI-ECG as a stand-alone decision maker. We expect that future advances of the AI-ECG model, including VHD prediction, will refine the performance of AI-ECG in preoperative risk stratification before noncardiac surgery.

Group 5 provides meaningful insights. Although this group demonstrated no abnormal findings in preoperative cardiovascular tests, it exhibited the highest rate of composite outcomes among all groups. A large proportion of these cases had undergone prior open-heart surgery, coronary artery bypass grafting, PCI, or stable coronary artery disease. Given the well-recognized elevated perioperative risk in these populations, repeated cardiovascular testing with unchanged results is unlikely to affect intraoperative or postoperative management. Therefore, combined with delicate history-taking about their symptoms, AI-ECG might be able to replace low-yield repeated examinations in clinically stable patients with a history of cardiac surgery or PCI. In addition, conditions overlooked in the preoperative assessment, such as atrial fibrillation or underlying myocardial disease, may have contributed to the adverse outcomes. These factors highlight potential novel targets for reducing postoperative complications beyond the established strategies.

### Potential Utility of AI-ECG Risk Stratification

Adding AI-ECG risk stratification to the model, incorporating age and RCRI, suggested potential for better identification of low-risk cases beyond age, yielding an improvement in the NRI and IDI. This improvement was largely driven by the effective identification of nonevents, which may facilitate more targeted resource allocation in low-risk individuals. Moreover, high-risk classification by AI-ECG was consistently associated with higher postoperative adverse event rates across diverse surgical subgroups in sensitivity analyses and in the PSM cohort. Clinically, however, the utility of AI-ECG–based screening may be greatest in specific settings, particularly in low-risk or emergency surgery where comprehensive preoperative evaluation is limited.

Low-risk patients are potential beneficiaries of AI-ECG risk stratification. Although current guidelines do not recommend performing additional tests in patients undergoing low-risk surgery not otherwise indicated by corresponding symptoms and/or signs of heart disease [5,6], a substantial number of low-risk surgeries are preceded by preoperative cardiovascular tests in the real world [24]. In our study, cases classified as low-risk (ie, having RCRI scores of 0 or 1 and involving low-risk surgeries by ESC surgical risk category) had very low composite outcome rates with high RR (Table 2). Namely, an AI-ECG could reduce low-yield tests, at least in patients with low surgical risk and low-risk AI-ECG. Therefore, integrating AI-ECG with established risk tools may outline patients who benefit from preoperative cardiovascular testing while curtailing the waste of medical resources.

Emergency or urgent surgeries could also benefit from an AI-ECG, in which the time for the preoperative workup is usually limited. As the risk of delaying surgery generally exceeds the benefit of information from preoperative examinations, risk assessment has been omitted for time-sensitive surgery [28]. However, the risk of developing composite outcomes in emergency surgeries is much higher than that in elective surgeries. If we could obtain information about the patient’s critical cardiac condition without delay, the results might change with intensive monitoring and delicate postoperative care. Among emergency surgery cases, the risk of the composite end point was much lower in the low-risk AI-ECG group (1.6%) than in the high-risk AI-ECG group (8.5%). Therefore, medical resources for postoperative care can be reassigned according to the rapid risk classification by AI-ECG, particularly in resource-limited settings.

### Potential for Health Care Resource Optimization

Beyond clinical outcomes, preoperative risk stratification using AI-ECG may offer meaningful implications for health care resource usage. In our cohort, 5458 cases underwent advanced cardiovascular testing, whereas only 3536 were classified as high-risk by AI-ECG. Theoretically, optimal integration of this tool could have decreased additional testing in up to 35.2% (1922/5458) of this population. However, because patient symptoms, signs, and functional capacity were not considered in this study, this reduction represents an absolute theoretical maximum and requires cautious interpretation. Furthermore, this estimation assumes complete clinician adherence to the AI-ECG recommendations, which may be difficult to achieve in real-world clinical practice due to defensive medicine, patient anxiety, and clinician intuition. These behavioral and systemic barriers would likely limit the actual reduction in medical resource usage. Nevertheless, it underscores its potential to optimize the medical resource allocation conceptually. Even when downstream cost analysis was performed under the exploratory assumption that cases in group 4 would involve advanced cardiovascular imaging in proportions similar to the overall tested cohort, AI-ECG–guided risk stratification continued to demonstrate favorable cost characteristics.

However, these findings should be interpreted in the context of each country’s health care system, including the costs of individual tests, waiting times, and insurance coverage. In South Korea, where universal health insurance facilitates easy access to diagnostic testing, physicians may prescribe more tests than those recommended by the guidelines, potentially leading to an overestimation of costs. Furthermore, although diagnostic costs for group 4 were partially included, downstream costs related to clinical outcomes, including beneficial or adverse events, were not included in this study. Applying the mean test frequency of the entire tested cohort to estimate the costs for group 4 also potentially results in an underestimation of their expected expenditure. Therefore, this exploratory cost analysis is not sufficient to prove the economic benefit of integrating the AI-ECG into preoperative evaluation, and a formal prospective cost-effectiveness analysis is necessary. Nevertheless, given its long-standing role in preoperative assessment, the ability of AI-ECG to refine risk stratification and reliably identify low-risk surgical candidates without additional expense may represent a meaningful advantage.

### Limitations

First, caution is warranted when expanding the use of preoperative risk stratification using AI-ECG into patients undergoing all noncardiac surgery. As a single-center retrospective analysis, our AI model is susceptible to overfitting and algorithm drift and lacks external validation in demographically distinct populations. Furthermore, within our specific cohort, the proportion of low-risk surgeries was relatively high, and the overall mortality rate was lower than that reported in previous studies. Additionally, the stand-alone PPV of 2.9% is partly depressed because several AI-ECG scores (eg, pulmonary hypertension or pericardial effusion) flag structural conditions that do not necessarily culminate in mortality or unplanned PCI. Consequently, the low PPV reinforces the role of the AI-ECG as a triage tool, rather than a definitive stand-alone diagnostic test. Moreover, as our primary end point included all-cause mortality alongside unplanned PCI, it is not a fully cardiovascular-specific metric and provides a relatively indirect assessment of a cardiovascular screening tool. Restricting mortality to inpatient records may have also missed out-of-hospital deaths, potentially underestimating the true 30-day mortality rate. Additionally, because unplanned PCI primarily captures plaque-rupture ischemic events, our end point likely missed medically managed postoperative cardiac morbidities, such as myocardial injury after noncardiac surgery or type 2 myocardial infarction, potentially underestimating their true burden. Furthermore, the unique insurance system in South Korea may have influenced the frequency and cost of preoperative testing, limiting the generalizability of cost-related findings. Lastly, the exclusion of cases without a recent preoperative ECG yielded an inherently risk-enriched cohort; thus, the AI-ECG is intended to triage patients who already meet clinical indications for baseline testing, rather than serving as a universal screening tool. Accordingly, the findings may not be directly generalizable to all cases of noncardiac surgery.

Second, the thresholds defining high- and low-risk categories were derived during the original model-development stage and were not recalibrated for the present largely asymptomatic preoperative cohort. To mitigate potential miscalibration, we applied a conservative composite-negative definition of low-risk, classifying cases as low-risk only when all cardiovascular disease-specific QCG scores were below their respective thresholds. While this approach prioritizes safety and minimizes false reassurance, it inevitably elevates the cumulative false-positive rate and may obscure intermediate levels of risk. Future studies incorporating recalibration or graded risk categories may further improve clinical applicability.

Third, our retrospective design introduces inherent temporal and selection biases. As we defined the index ECG as the one closest to surgery within a 30-day window to optimally reflect the most recent preoperative state, unrecorded physiological fluctuations could occur during this interval between ECG and surgery. Furthermore, this design allowed for a temporal overlap where diagnostic imaging may have been performed before the index ECG. Thus, we could not fully account for the possible impact of medical optimization following abnormal testing before the index ECG. Although noninvasive imaging does not alter cardiac electrophysiology, a true clinical screening tool must temporally precede the decision to test. Moreover, although AI-ECG risk stratification was associated with postoperative outcomes, it remains unclear whether its use, followed by appropriate interventions, would improve clinical outcomes or reduce overall expenses. As preoperative testing was not randomly assigned and reflects clinical judgment, the observed associations should not be interpreted causally. Additionally, although we performed PSM analysis to balance the clinical likelihood of undergoing advanced cardiovascular testing, the matched cohort contains 1019 AI-ECG high-risk cases in the tested arm (groups 5 and 6 combined) vs 747 in the nontested arm (group 4). Therefore, a residual imbalance in the distribution of AI-ECG high-risk cases remained between the matched arms. Furthermore, baseline demographic differences remained unadjusted in the main analysis and might influence the observed event rates. Thus, the predictive value of AI-ECG risk stratification might be partially driven by unadjusted variables, such as advancing age and cumulative comorbidities. Nevertheless, it should be noted that our supplementary analyses demonstrated its independent prognostic performance even after adjusting for age and RCRI, suggesting that the AI-ECG provides unique prognostic value beyond advanced age and comorbidities. Future prospective studies should contextualize the stand-alone value of the AI-ECG against comprehensive calculators incorporating age, such as ACS National Surgical Quality Improvement Program.

Fourth, the definitions used for conventional cardiovascular testing may have influenced the comparative diagnostic yields of cardiovascular tests. Stress testing and biomarkers, such as high-sensitivity troponin and natriuretic peptides, were not included as preoperative cardiovascular tests, which may have led to an underestimation of the scope of conventional testing and potentially inflated the stand-alone value of the AI-ECG by ignoring biochemical risk clearance. Additionally, although resting echocardiography is a mainstay of preoperative evaluation in clinical practice, its ability to predict acute coronary ischemic events that necessitate unplanned PCI is limited. This modality–end point mismatch should be considered when interpreting the conventional testing as low-yield. Conversely, defining a positive anatomical finding as ≥50% coronary stenosis might have artificially inflated the diagnostic yield of cardiovascular testing. Using strictly actionable thresholds could widen the comparative efficiency between the AI-ECG tool and conventional testing.

Finally, as clinical symptoms, signs, and functional capacity were not incorporated in this study, further evaluation remains necessary for patients with suspected but undiagnosed cardiac disease, regardless of AI-ECG classification. AI-ECG–based risk stratification cannot replace history-taking or physical examination in the decision-to-test process. Additionally, omitting echocardiography in patients with murmurs solely based on a low-risk AI-ECG score could lead to significant missed pathology. This underscores the role of the AI-ECG as an adjunctive screening tool rather than a stand-alone decision maker.

To overcome these limitations, prospective multicenter randomized controlled trials should be conducted for a comprehensive assessment of whether the AI-ECG can improve postoperative outcomes and reduce the cost related to preoperative cardiovascular tests in noncardiac surgery.

### Conclusions

In conclusion, within the limitations of this retrospective study, a low-risk classification by multitask AI-ECG was associated with a low incidence of postoperative adverse events. By integrating AI-ECG with established risk-stratification tools, we identified a subgroup of cases in which advanced preoperative cardiovascular testing demonstrated limited yield. These exploratory findings suggest that AI-ECG may serve as a potentially resource-optimizing screening adjunct to present preoperative assessment strategies in noncardiac surgery. However, prospective validation is required to formally establish its safety and cost-effectiveness.

## Data Availability

The datasets analyzed during this study are not publicly available due to institutional restrictions but are available from the corresponding author upon reasonable request.

## Appendix

Multimedia Appendix 1 Data for artificial intelligence–enabled electrocardiography (AI-ECG)–based preoperative risk stratification.

## Abbreviations

ACS: acute coronary syndrome
AI: artificial intelligence
CAG: coronary angiography
CCTA: coronary computed tomography angiography
ECG: electrocardiography
ESC: European Society of Cardiology
GEE: generalized estimating equation
IDI: integrated discrimination improvement
NRI: net reclassification improvement
PCI: percutaneous coronary intervention
PPV: positive predictive value
PSM: propensity score matching
QCG: quantitative score for electrocardiography
RCRI: Revised Cardiac Risk Index
RR: relative risk
SPECT: single-photon emission computed tomography
STROBE: Strengthening the Reporting of Observational Studies in Epidemiology
VHD: valvular heart disease
