# Supplementary material for: Multitask Artificial Intelligence–Based Electrocardiogram Tool for Preoperative Cardiac Testing in Noncardiac Surgery: Retrospective Cohort Study of Health Care Utilization and Costs
Source: J Med Internet Res. 2026 Jun 17;28:e90099. doi: 10.2196/90099 (PMC13324315; doi:10.2196/90099)

**Multitask Artificial Intelligence–Based Electrocardiogram Tool for Preoperative Cardiac Testing in Noncardiac Surgery: Retrospective Cohort Study of Health Care Utilization and Costs**

**Table S1.** The list of QCG scores used to define the risk by AI-enabled ECG.

**Table S2.** Characteristics according to the preoperative cardiovascular test groups.

**Table S3.** Analysis for exploratory composite outcome (all-cause death, unplanned percutaneous coronary intervention [PCI], or prolonged mechanical ventilation (≥3 days)).

**Table S4.** Comparison of preoperative cardiovascular (CV) tests and the results stratified by study groups.

**Table S5.** Sensitivity analyses of postoperative adverse outcomes (death or unplanned percutaneous coronary intervention) by AI-ECG risk stratification across surgical subgroups.

**Table S6.** The patient-level sensitivity analysis restricted to each patient’s first surgery (N=41,218): the incidence, relative risk, and relative difference of the composite outcome across ESC surgical risk category and RCRI strata by AI-ECG risk stratification.

**Table S7.** Baseline characteristics of the study population before and after propensity score matching for advanced cardiovascular testing.

**Table S8.** Clinical outcomes according to AI-ECG risk stratification and advanced cardiovascular testing in the propensity score-matched cohort.

**Figure S1.** Study flow.

**Figure S2.** The distribution of QCG scores.

**Figure S3.** Composite outcome rates according to the reclassification using AI-ECG.

**Figure S4.** (A) Logistic regression analysis for the composite outcome, including age, RCRI categories and AI-ECG risk stratification.
(B) Receiver operating characteristic (ROC) curves and area under the ROC curve (AUROC) values among different perioperative risk models (Model 1, blue; Model 2, red).
(C) Reclassification and discrimination improvement by adding AI-ECG to the clinical risk model (RCRI).
(D) Calibration plot of the multivariable logistic regression model (Model 2).
(E) Precision-Recall (PR) curve for the multivariable model (Model 2).

**Figure S5.** The explainable causes of surgery without further preoperative cardiovascular testing in group 4.

**Figure S6.** The representative case with abnormal coronary angiography (CAG) results requiring revascularization before proceeding to surgery.

**Table S1.** The list of QCG scores used to define the risk by AI-enabled ECG**.**

| QCG scores | Definition | Youden threshold | Sensitivity | Specificity |
| --- | --- | --- | --- | --- |
| ACS | Risk of acute coronary syndrome (STEMI, NSTEMI, and UA) | 19.7 | 76.9% | 94.2% |
| STEMI | Risk of ST-segment elevation myocardial infarction developed within 12 hours | 6.6 | 97.5% | 99.0% |
| Myocardial injury | Risk of elevated cardiac troponin | 19.1 | 76.2% | 89.7% |
| Pulmonary edema | Risk of pulmonary edema | 18.1 | 86.0% | 86.5% |
| Pericardial effusion | Risk of large amount of pericardial effusion | 24.6 | 85.3% | 91.0% |
| LV dysfunction | Risk of LVEF < 40% | 30.1 | 90.4% | 87.8% |
| RV dysfunction | Risk of RV dysfunction | 26.0 | 91.6% | 81.5% |
| Pulmonary hypertension | Risk of moderate or severe pulmonary hypertension | 29.3 | 83.0% | 79.1% |

**Table S2.** Characteristics according to the preoperative cardiovascular test groups.

|  | No Advanced CV Imaging (N=40,677) | Negative-test (N=4,566) | Positive-test (N=892) | p |
| --- | --- | --- | --- | --- |
| Demographics and comorbidities |  |  |  |  |
| Age (mean (SD)) | 54.80 (15.83) | 69.24 (11.92) | 71.71 (11.23) | <0.001 |
| Male sex (%) | 17745 (43.6) | 2219 (48.6) | 597 (66.9) | <0.001 |
| DM (%) | 4630 (11.4) | 968 (21.2) | 306 (34.3) | <0.001 |
| DM treated with insulin (%) | 411 (1.0) | 159 (3.5) | 62 (7.0) | <0.001 |
| Hypertension (%) | 8492 (20.9) | 1829 (40.1) | 438 (49.1) | <0.001 |
| Previous heart failure (%) | 110 (0.3) | 49 (1.1) | 251 (28.1) | <0.001 |
| Previous ischemic heart disease (%) | 990 (2.4) | 345 (7.6) | 437 (49.0) | <0.001 |
| Previous cerebrovascular accident (%) | 1164 (2.9) | 260 (5.7) | 101 (11.3) | <0.001 |
| Smoking (%) |  |  |  |  |
| Nonsmoker | 24730 (60.8) | 3217 (70.5) | 489 (54.8) | <0.001 |
| Smoker | 8299 (20.4) | 1234 (27.0) | 371 (41.6) |  |
| Unknown | 7648 (18.8) | 115 (2.5) | 32 (3.6) |  |
| Creatinine (median [IQR]) | 0.89 (0.96) | 1.07 (1.25) | 1.65 (1.95) | <0.001 |
| Creatinine ≥ 2.0 mg/dl (%) | 1028 (2.5) | 243 (5.3) | 143 (16.0) | <0.001 |
| Surgery-related |  |  |  |  |
| Emergency surgery (%) | 2619 (6.4) | 132 (2.9) | 55 (6.2) | <0.001 |
| Anesthesia (%) |  |  |  |  |
| General anesthesia | 29263 (71.9) | 3490 (76.4) | 633 (71.0) | <0.001 |
| Spinal/Epidural anesthesia | 3387 (8.3) | 792 (17.3) | 93 (10.4) |  |
| Monitored anesthesia care | 8027 (19.7) | 284 (6.2) | 166 (18.6) |  |
| ESC surgical risk category (%) |  |  |  |  |
| Low | 23245 (57.1) | 1286 (28.2) | 294 (33.0) | <0.001 |
| Intermediate | 13561 (33.3) | 2513 (55.0) | 318 (35.7) |  |
| High | 3871 (9.5) | 767 (16.8) | 280 (31.4) |  |
| RCRI (%) |  |  |  |  |
| 0 | 26512 (65.2) | 2111 (46.2) | 115 (12.9) | <0.001 |
| 1 | 13127 (32.3) | 2005 (43.9) | 336 (37.7) |  |
| 2 | 885 (2.2) | 384 (8.4) | 293 (32.8) |  |
| 3 | 123 (0.3) | 59 (1.3) | 126 (14.1) |  |
| 4 | 25 (0.1) | 7 (0.2) | 18 (2.0) |  |
| 5 | 5 (0.0) | 0 (0.0) | 4 (0.4) |  |
| ASA classification (%) |  |  |  |  |
| 1 | 13568 (33.4) | 245 (5.4) | 1 (0.1) | <0.001 |
| 2 | 21451 (52.7) | 2545 (55.7) | 134 (15.0) |  |
| 3 | 5176 (12.7) | 1595 (34.9) | 561 (62.9) |  |
| 4 | 401 (1.0) | 170 (3.7) | 194 (21.7) |  |
| 5 | 81 (0.2) | 11 (0.2) | 2 (0.2) |  |
| QCG scores (median [IQR]) |  |  |  |  |
| ACS | 3.33 (6.28) | 6.01 (8.26) | 14.93 (17.49) | <0.001 |
| STEMI | 0.21 (1.65) | 0.52 (2.35) | 3.38 (9.22) | <0.001 |
| Myocardial injury | 3.50 (6.27) | 6.37 (8.40) | 16.61 (17.87) | <0.001 |
| Pulmonary edema | 2.29 (5.75) | 5.41 (9.08) | 16.64 (18.32) | <0.001 |
| Pericardial effusion | 0.78 (3.77) | 2.01 (6.62) | 5.10 (9.75) | <0.001 |
| Left ventricular dysfunction | 1.16 (6.08) | 2.46 (7.87) | 19.13 (27.20) | <0.001 |
| Right ventricular dysfunction | 1.23 (4.86) | 2.98 (7.92) | 11.47 (16.88) | <0.001 |
| Pulmonary hypertension | 1.33 (4.56) | 3.55 (7.73) | 11.06 (15.05) | <0.001 |

Abbreviations: CV, cardiovascular; SD, standard deviations; DM, diabetes mellitus, IQR, interquartile range; ESC, European Society of Cardiology; RCRI, Revised Cardiac Risk Index; ASA, American Society of Anesthesiologists; ACS, acute coronary syndrome; STEMI, ST-segment elevation myocardial infarction.

**Table S3.** Analysis for exploratory composite outcome (all-cause death, unplanned percutaneous coronary intervention [PCI], or prolonged mechanical ventilation (≥3 days)).

| Death, PCI, MV | Group | N | Events | Event rate | Group | N | Events | Event rate | Relative risk |
| --- | --- | --- | --- | --- | --- | --- | --- | --- | --- |
| All patients | AI Low-risk | 42599 | 265 | 0.62 | AI High-risk | 3536 | 212 | 6.00 | 9.64 |
|  | Group 1 | 38348 | 213 | 0.56 | Group 4 | 2329 | 143 | 6.14 |  |
|  | Group 2 | 3868 | 41 | 1.06 | Group 5 | 698 | 44 | 6.3 |  |
|  | Group 3 | 383 | 11 | 2.87 | Group 6 | 509 | 25 | 4.91 |  |
| Subgroup 1:  Major surgery under general anesthesia | AI Low-risk | 16467 | 244 | 1.48 | AI High-risk | 1686 | 175 | 10.4 | 7.00 |
|  | Group 1 | 14106 | 195 | 1.38 | Group 4 | 1029 | 119 | 11.6 |  |
|  | Group 2 | 2125 | 38 | 1.79 | Group 5 | 395 | 34 | 8.61 |  |
|  | Group 3 | 236 | 11 | 4.66 | Group 6 | 262 | 22 | 8.4 |  |
| Subgroup 2:  Elective surgery | AI Low-risk | 40262 | 114 | 0.283 | AI High-risk | 3067 | 92 | 3.00 | 10.59 |
|  | Group 1 | 36118 | 73 | 0.20 | Group 4 | 1940 | 48 | 2.47 |  |
|  | Group 2 | 3780 | 32 | 0.85 | Group 5 | 654 | 26 | 3.98 |  |
|  | Group 3 | 364 | 9 | 2.47 | Group 6 | 473 | 18 | 3.81 |  |
| Subgroup 3:  Emergency surgery | AI Low-risk | 2337 | 151 | 6.46 | AI High-risk | 469 | 120 | 25.6 | 3.96 |
|  | Group 1 | 2230 | 140 | 6.28 | Group 4 | 389 | 95 | 24.4 |  |
|  | Group 2 | 88 | 9 | 10.2 | Group 5 | 44 | 18 | 40.9 |  |
|  | Group 3 | 19 | 2 | 10.5 | Group 6 | 36 | 7 | 19.4 |  |

**Table S4.** Comparison of preoperative cardiovascular (CV) tests and the results stratified by study groups.

|  | AI-ECG low-risk | | AI-ECG high-risk | |  |
| --- | --- | --- | --- | --- | --- |
|  | Group 2  (N=3868) | Group 3  (N=383) | Group 5  (N=698) | Group 6  (N=509) | p |
| Any preoperative CV test performed (%) | 3868 (100.0) | 383 (100.0) | 698 (100.0) | 509 (100.0) | NA |
| Abnormal preoperative CV test (%) | 0 (0.0) | 383 (100.0) | 0 (0.0) | 509 (100.0) | <0.001 |
| Echocardiography performed (%) | 3553 (91.9) | 289 (75.5) | 601 (86.1) | 365 (71.7) | <0.001 |
| Abnormal echocardiography results (%) | 0 (0.0) | 243 (84.1) | 0 (0.0) | 331 (90.7) | <0.001 |
| Moderate or severe valvular heart disease (%) |  | 114 (39.4) |  | 121 (33.2) |  |
| Severe valvular heart disease (%) |  | 19 (9.7) |  | 46 (12.6) |  |
| Severe valvular heart disease with LV ejection fraction <50% (%) |  | 1 (0.3) |  | 14 (3.8) |  |
| Multiple severe valvular heart disease (%) |  | 0 (0.0) |  | 6 (1.6) |  |
| Regional wall motion abnormality (%) | 0 (0.0) | 130 (45.0) | 0 (0.0) | 244 (68.2) | <0.001 |
| Heart failure (%) | 0 (0.0) | 54 (18.7) | 0 (0.0) | 171 (47.1) | <0.001 |
| LV ejection fraction (%) | 63.3 [60.0, 66.7] | 59.7 [52.3, 65.2] | 61.9 [58.5, 66.0] | 51.1 [41.8, 59.6] | <0.001 |
| Coronary angiography performed (%) | 77 (2.0) | 106 (27.7) | 54 (7.7) | 233 (45.8) | <0.001 |
| Abnormal coronary angiography results (%) | 0 (0.0) | 76 (71.7) | 0 (0.0) | 197 (84.5) | <0.001 |
| 3-vessel disease (%) |  | 26 (24.5) |  | 78 (33.5) |  |
| >70% stenosis (%) |  | 62 (58.4) |  | 166 (71.2) |  |
| Revascularization before surgery (%) |  | 13 (12.3) |  | 76 (32.6) |  |
| Coronary CT angiography performed (%) | 284 (7.3) | 127 (33.2) | 68 (9.7) | 100 (19.6) | <0.001 |
| Abnormal coronary CT angiography results (%) | 0 (0.0) | 99 (79.8) | 0 (0.0) | 66 (68.8) | <0.001 |
| SPECT performed (%) | 128 (3.3) | 22 (5.7) | 51 (7.3) | 44 (8.6) | <0.001 |
| Abnormal SPECT results (%) | 0 (0.0) | 3 (13.6) | 0 (0.0) | 4 (9.1) | <0.001 |

**Table S5.** Sensitivity analyses of postoperative adverse outcomes (death or unplanned percutaneous coronary intervention) by AI-ECG risk stratification across surgical subgroups.

|  | Group | N | Events | Event rate | Group | N | Events | Event rate | Relative risk |
| --- | --- | --- | --- | --- | --- | --- | --- | --- | --- |
| Subgroup 1:  Major surgery under general anesthesia | AI Low-risk | 16467 | 68 | 0.41 | AI High-risk | 1686 | 69 | 4.09 | 9.91 |
|  | Group 1 | 14106 | 55 | 0.39 | Group 4 | 1029 | 42 | 4.08 |  |
|  | Group 2 | 2125 | 12 | 0.56 | Group 5 | 395 | 16 | 4.05 |  |
|  | Group 3 | 236 | 1 | 0.42 | Group 6 | 262 | 11 | 4.20 |  |
| Subgroup 2:  Elective surgery | AI Low-risk | 40262 | 41 | 0.10 | AI High-risk | 3067 | 61 | 1.99 | 19.53 |
|  | Group 1 | 36118 | 28 | 0.08 | Group 4 | 1940 | 35 | 1.8 |  |
|  | Group 2 | 3780 | 13 | 0.34 | Group 5 | 654 | 17 | 2.6 |  |
|  | Group 3 | 364 | 0 | 0.00 | Group 6 | 473 | 9 | 1.9 |  |
| Subgroup 3:  Emergency surgery | AI Low-risk | 2337 | 38 | 1.63 | AI High-risk | 469 | 40 | 8.53 | 5.25 |
|  | Group 1 | 2230 | 36 | 1.61 | Group 4 | 389 | 29 | 7.46 |  |
|  | Group 2 | 88 | 1 | 1.14 | Group 5 | 44 | 7 | 15.9 |  |
|  | Group 3 | 19 | 1 | 5.26 | Group 6 | 36 | 4 | 11.1 |  |

**Table S6.** The patient-level sensitivity analysis restricted to each patient’s first surgery (N=41,218): the incidence, relative risk, and relative difference of the composite outcome across ESC surgical risk category and RCRI strata by AI-ECG risk stratification.

|  |  | Incidence of composite outcomes | | | |
| --- | --- | --- | --- | --- | --- |
|  | Risk group | AI-ECG  low-risk (%) (95% CI) | AI-ECG  high-risk (%)  (95% CI) | RR  (95% CI) | RD %p  (95% CI) |
| Surgical Emergency | Elective | 0.09 (0.06–0.12) | 1.69 (1.22–2.28) | 19.81 (12.48–31.45) | 1.61 (1.10–2.11) |
|  | Emergency | 1.72 (1.20–2.38) | 9.57 (6.80–13.01) | 5.57 (3.55–8.76) | 7.86 (4.83–10.88) |
| ESC surgical risk | Low | 0.01 (0.00–0.04) | 1.32 (0.76–2.14) | 92.65 (27.03–317.53) | 1.31 (0.67–1.96) |
|  | Intermediate | 0.30 (0.21–0.40) | 2.94 (2.01–4.15) | 9.93 (6.26–15.77) | 2.65 (1.62–3.67) |
|  | High | 0.62 (0.39–0.94) | 5.18 (3.55–7.28) | 8.30 (4.84–14.24) | 4.56 (2.76–6.36) |
| RCRI | 0–1 | 0.14 (0.11–0.19) | 2.12 (1.57–2.80) | 14.71 (10.01–21.60) | 1.98 (1.39–2.57) |
|  | 2+ | 1.28 (0.66–2.23) | 5.26 (3.55–7.47) | 4.10 (2.11–7.97) | 3.98 (1.98–5.98) |
| Total |  | 0.17 (0.13–0.22) | 2.73 (2.16–3.39) | 15.85 (11.45–21.95) | 2.56 (1.96–3.15) |

**Table S7.** Baseline characteristics of the study population before and after propensity score matching for advanced cardiovascular testing.

|  | Advanced CV test (N=5,122) | No advanced CV test (N=5,122) | Standardized mean difference (SMD) |
| --- | --- | --- | --- |
| Distance | 29.9 | 29.2 | 0.029 |
| *Demographics and comorbidities* |  |  |  |
| Age (mean (SD)) | 69.1 | 69.4 | 0.031 |
| Male sex (%) | 51.2 | 52.9 | 0.034 |
| Diabetes (%) | 22.6 | 22.8 | 0.006 |
| Hypertension (%) | 40.8 | 41.3 | 0.010 |
| Heart failure (%) | 3.1 | 2.0 | 0.050 |
| Ischemic heart disease (%) | 11.8 | 11.7 | 0.003 |
| Creatinine ≥2.0 mg/dl (%) | 6.5 | 7.1 | 0.022 |
| *Surgery-related* |  |  |  |
| Emergency surgery (%) | 3.4 | 4.0 | 0.031 |
| Anesthesia (%) |  |  |  |
| General anesthesia | 76.0 | 77.8 | 0.043 |
| Spinal/Epidural anesthesia | 15.6 | 13.9 | 0.048 |
| Monitored anesthesia care | 8.4 | 8.4 | 0.003 |
| ESC surgical risk category (%) |  |  |  |
| Low | 30.1 | 32.0 | 0.043 |
| Intermediate | 52.0 | 52.9 | 0.018 |
| High | 17.9 | 15.1 | 0.072 |
| RCRI (%) |  |  |  |
| 0 | 42.9 | 42.5 | 0.007 |
| 1 | 43.9 | 45.6 | 0.035 |
| 2 | 10.7 | 9.6 | 0.035 |
| 3 | 2.2 | 1.9 | 0.017 |
| 4 | 0.3 | 0.4 | 0.012 |
| 5 | 0.1 | 0.0 | 0.007 |
| ASA classification (%) |  |  |  |
| 1 | 4.8 | 4.7 | 0.006 |
| 2 | 52.1 | 50.4 | 0.035 |
| 3 | 38.0 | 40.7 | 0.056 |
| 4 | 4.9 | 4.0 | 0.037 |
| 5 | 0.2 | 0.3 | 0.004 |

**Table S8.** Clinical outcomes according to AI-ECG risk stratification and advanced cardiovascular testing in the propensity score-matched cohort.

| Group | AI-ECG group | CV test | Events (N) | Total (N) | Event rate (%) |
| --- | --- | --- | --- | --- | --- |
| 1 | AI-ECG low | No advanced CV test | 15 | 4375 | 0.34 |
| 2 | AI-ECG low | Advanced CV test - Negative | 13 | 3780 | 0.34 |
| 3 | AI-ECG low | Advanced CV test - Positive | 1 | 323 | 0.31 |
| 4 | AI-ECG high | No advanced CV test | 33 | 747 | 4.42 |
| 5 | AI-ECG high | Advanced CV test - Negative | 22 | 650 | 3.38 |
| 6 | AI-ECG high | Advanced CV test - Positive | 10 | 369 | 2.71 |

**Figure S1.** Study flow.


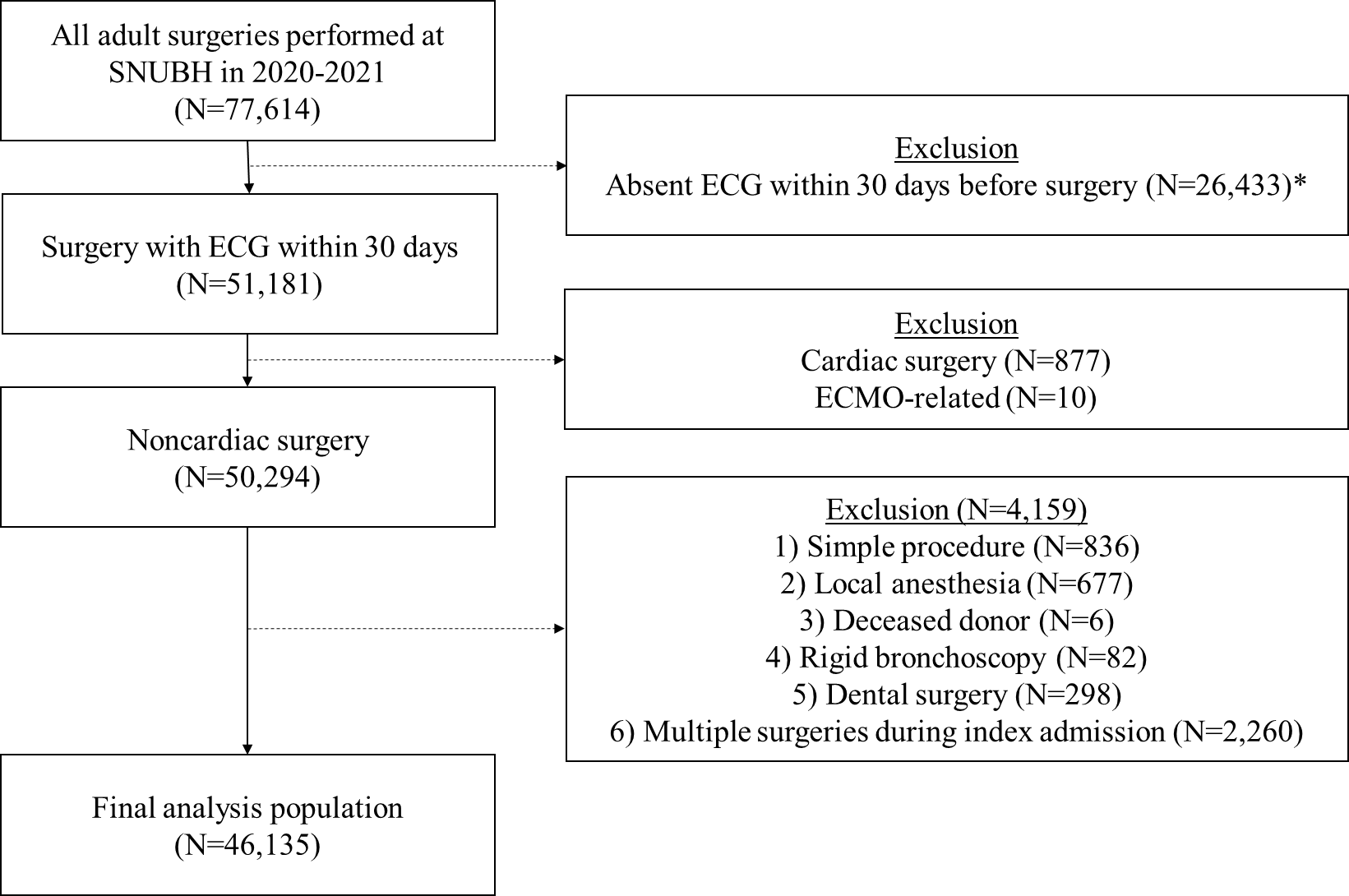


* For the 26 patients who underwent coronary revascularization, the index preoperative ECG was designated using a modified timeframe, although all 26 patients had at least one ECG performed within 30 days prior to surgery.

**Figure S2.** The distribution of QCG scores. (A) Histograms for the distribution of individual QCG scores. Red dashed line represents the optimal cutoffs for individual scores. (B) The distribution of QCG scores across the six study groups. Low-risk AI-ECG group was illustrated in light blue, while high-risk AI-ECG group with orange.

**(A)**


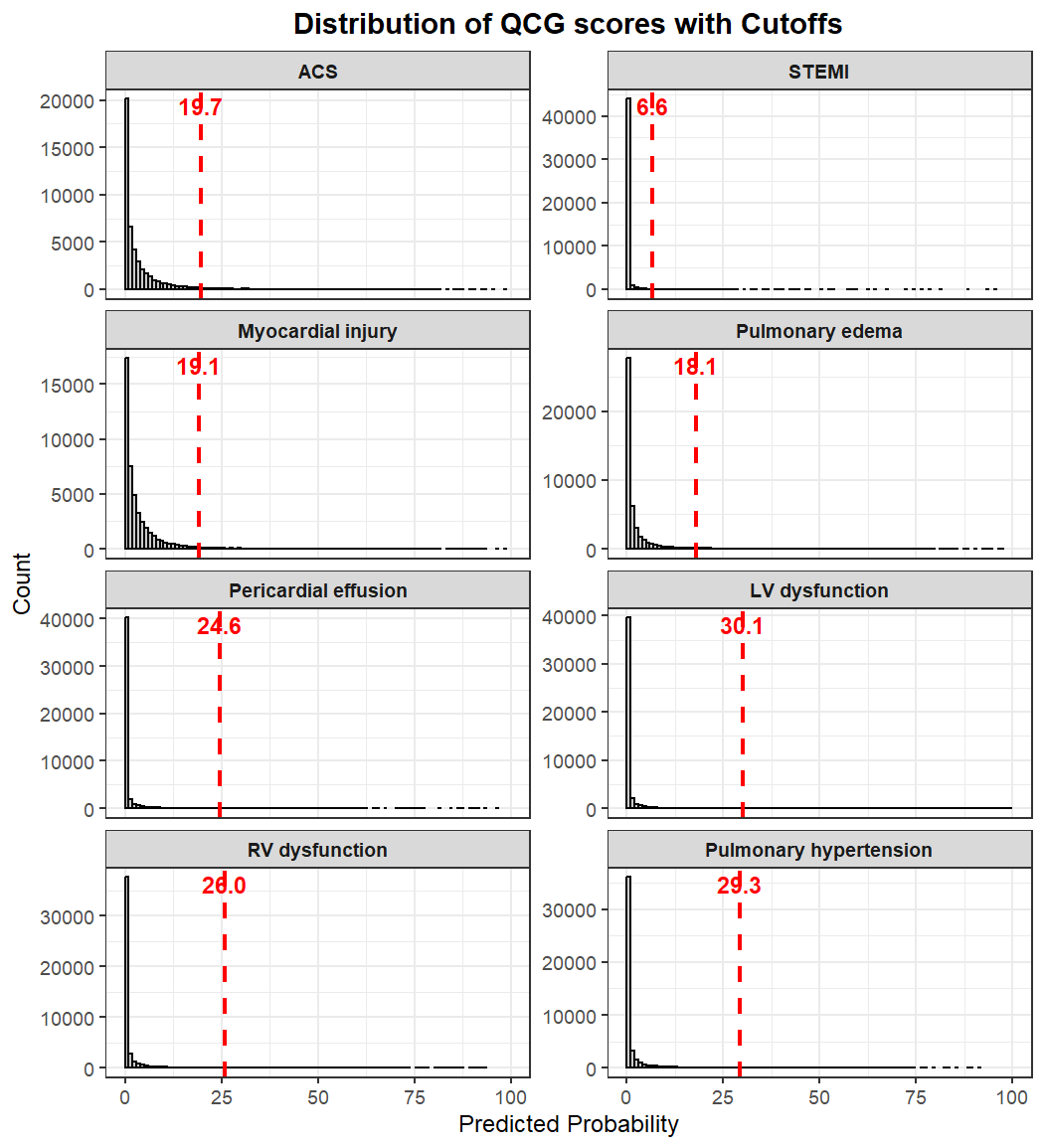


**(B)**


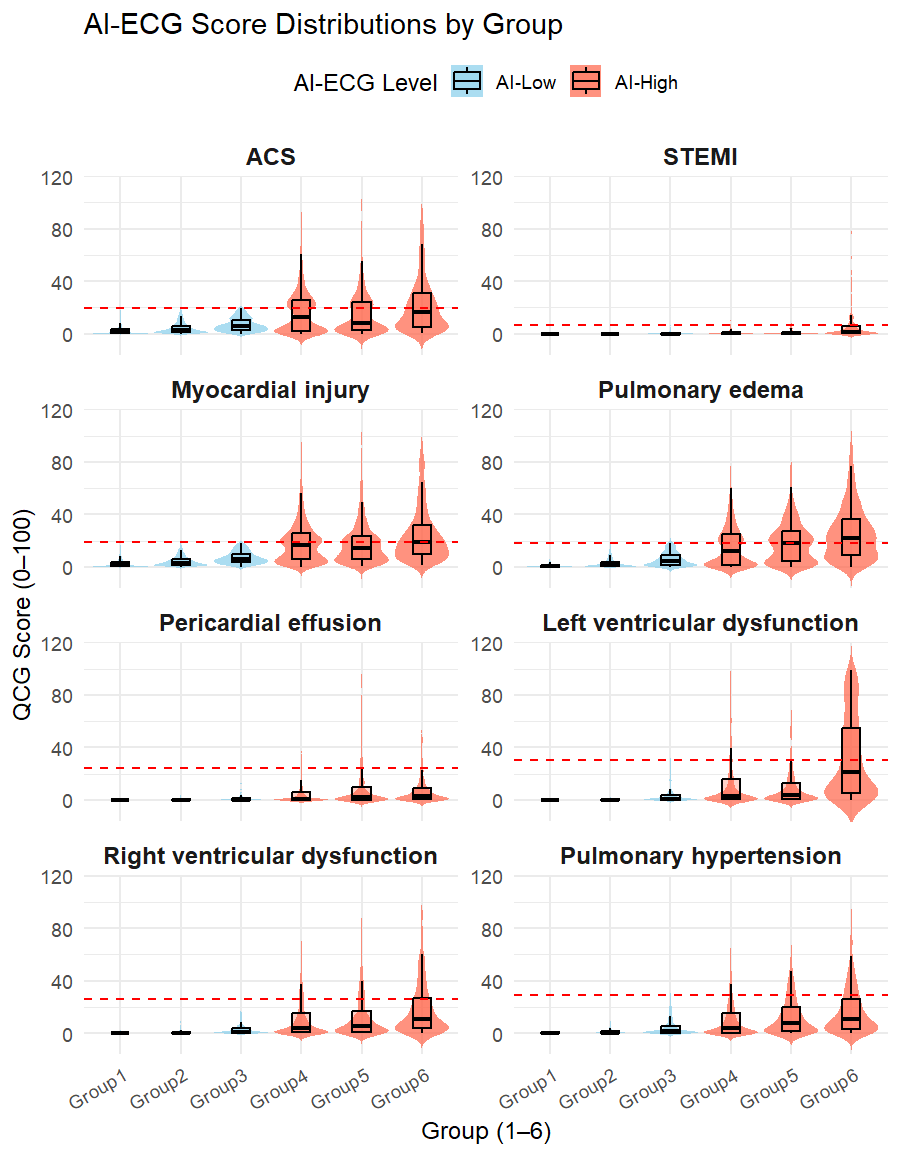


**Figure S3.** Composite outcome rates according to the reclassification using AI-ECG. (A) The European Society of Cardiology (ESC) surgical risk categories and (B) the Revised Cardiac Risk Index (RCRI) are reclassified by AI-ECG. Within each conventional surgical risk category, further risk stratification by AI-ECG demonstrates consistently higher composite outcome rates in the high-risk AI-ECG group than in the low-risk AI-ECG group.


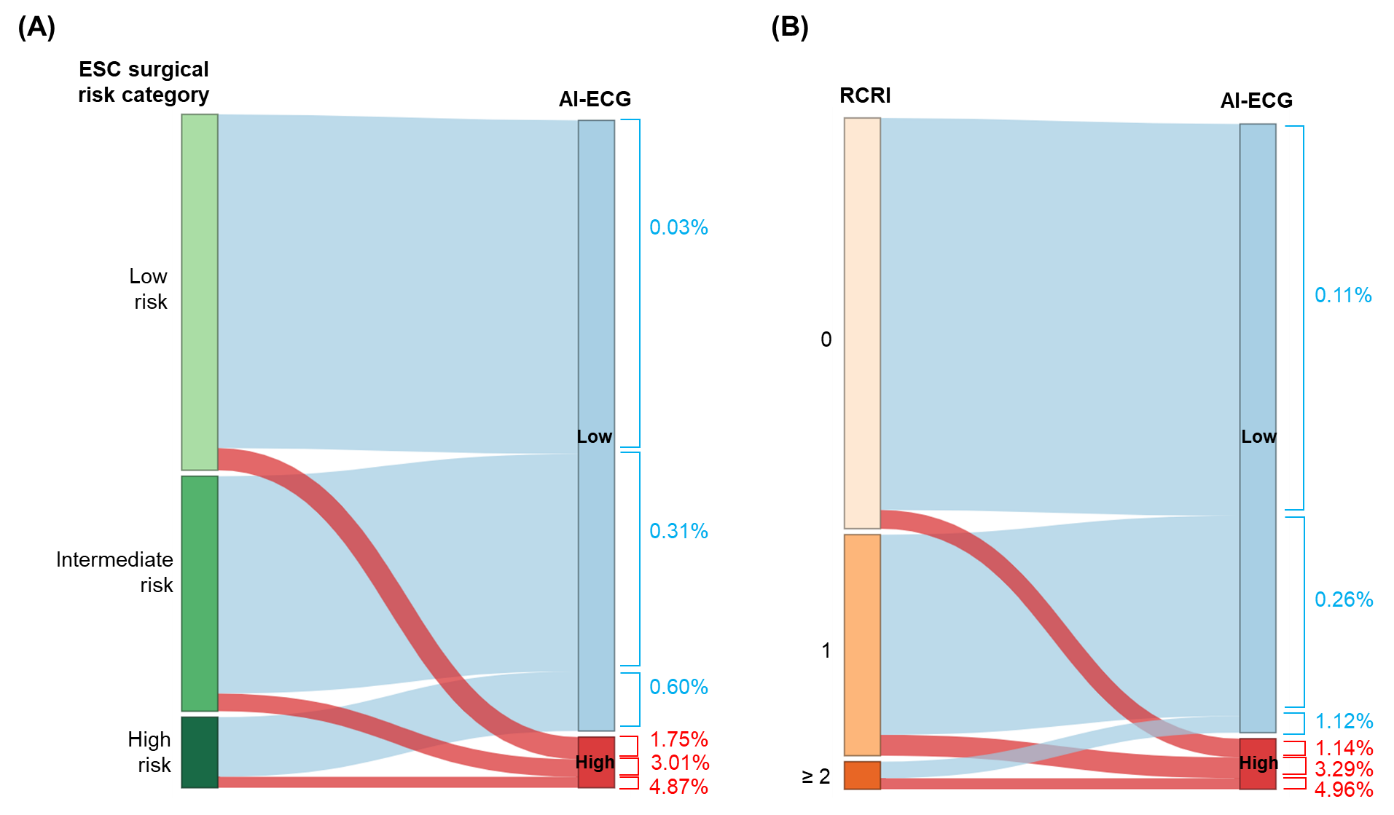


**Figure S4.** (A) Logistic regression analysis for the composite outcome, including age, RCRI categories and AI-ECG risk stratification. RCRI was modeled as an ordered categorical variable (0–1 vs ≥2). Odds ratios represent a per-category increase. (B) Receiver operating characteristic (ROC) curves and area under the ROC curve (AUROC) values among different perioperative risk models (Model 1, blue; Model 2, red). (C) Reclassification and discrimination improvement by adding AI-ECG to the clinical risk model (RCRI). (D) Calibration plot of the multivariable logistic regression model (Model 2). The x-axis represents the predicted probability of composite outcomes, and the y-axis represents the actual observed frequency. The dashed line indicates the ideal performance of a perfectly calibrated model. The solid line represents the bias-corrected performance of the model using 100 bootstrap resamples. C.L. means the 95% confidence limit. (E) Precision-Recall (PR) curve for the multivariable model (Model 2). The area under the PR curve (AUPRC) was 0.038.

**(A)**

|  | **Model 1**  **(age + RCRI)** | | **Model 2**  **(age + RCRI + AI-ECG high risk)** | |
| --- | --- | --- | --- | --- |
|  | OR (95% CI) | p | OR (95% CI) | p |
| Age | 1.05 [1.04–1.07] | <0.001 | 1.04 [1.02–1.05] | <0.001 |
| RCRI 0 | Reference |  | Reference |  |
| RCRI 1 | 3.28 [2.29–4.76] | <0.001 | 2.61 [1.81–3.81] | <0.001 |
| RCRI 2+ | 10.4 [6.84–15.80] | <0.001 | 4.67 [3.01–7.28] | <0.001 |
| AI-ECG high risk |  |  | 7.38 [5.30–10.80] | <0.001 |

**(B)**

**
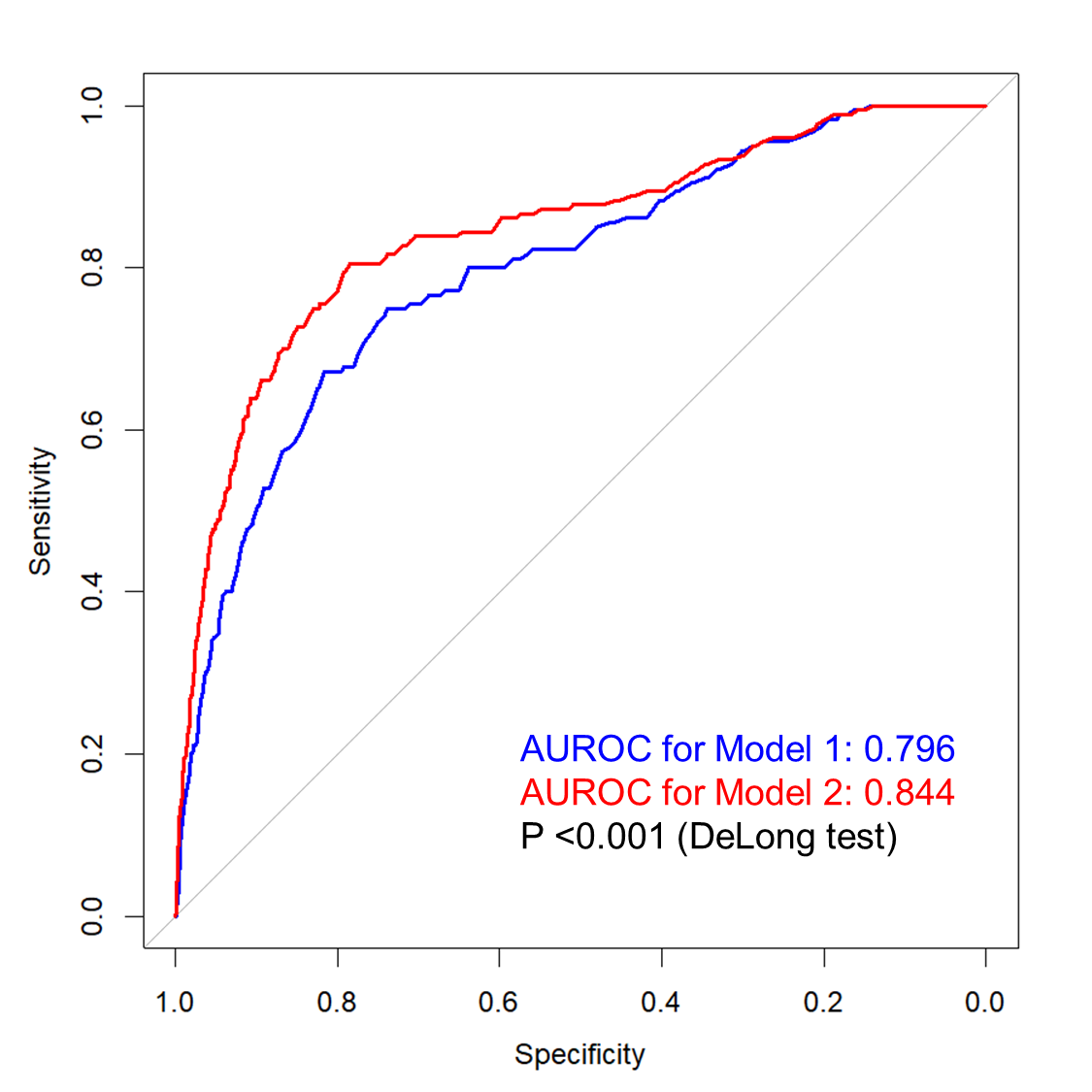
**

**(C)**

|  | Index | 95% CI | p value |
| --- | --- | --- | --- |
| Net Reclassification Index (NRI) | 0.675 | [0.530–0.820] | <0.001 |
| NRI for events | 0.144 | [-0.0001–0.289] | 0.050 |
| NRI for non-events | 0.530 | [0.523–0.538] | <0.001 |
| IDI | 0.012 | [0.009–0.014] | <0.001 |

**(D)**

**
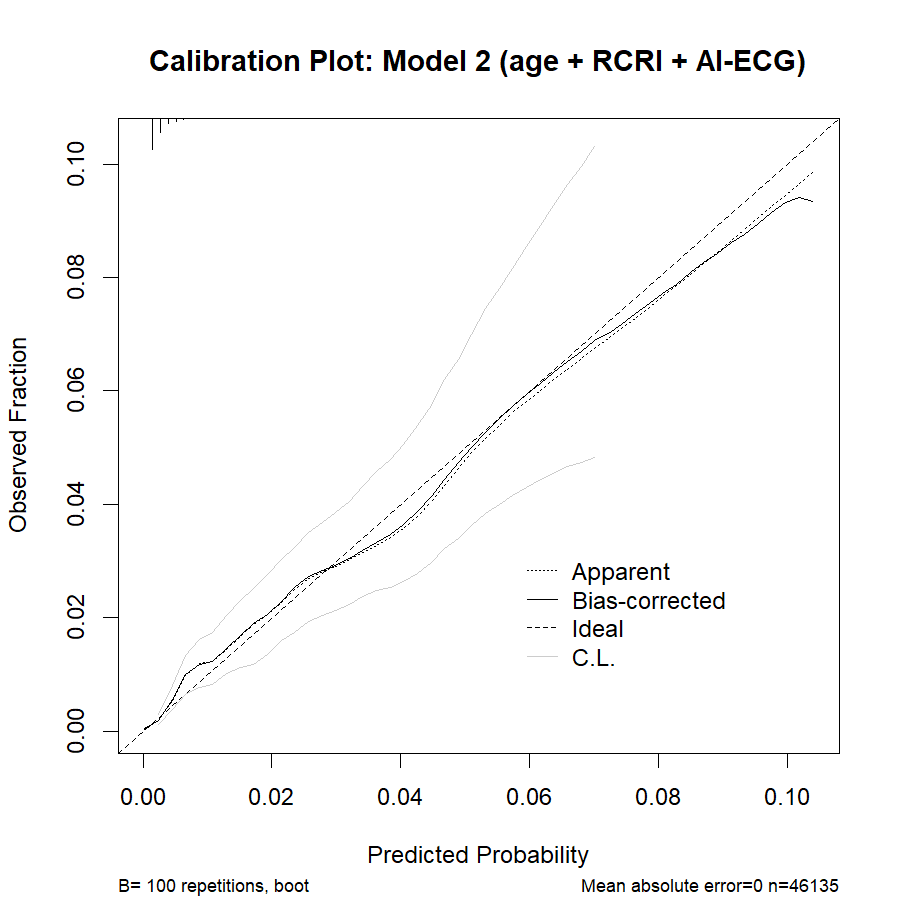
**

**(E)**

**
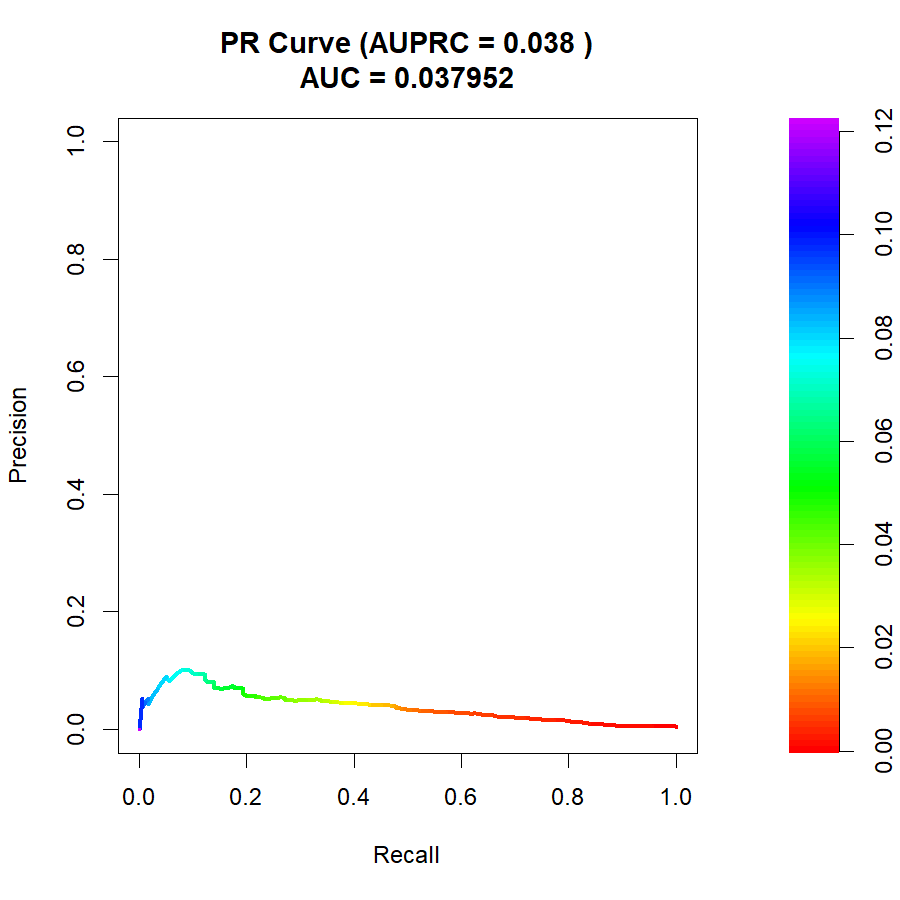
**

**Figure S5.** The explainable causes of surgery without further preoperative cardiovascular testing in Group 4.


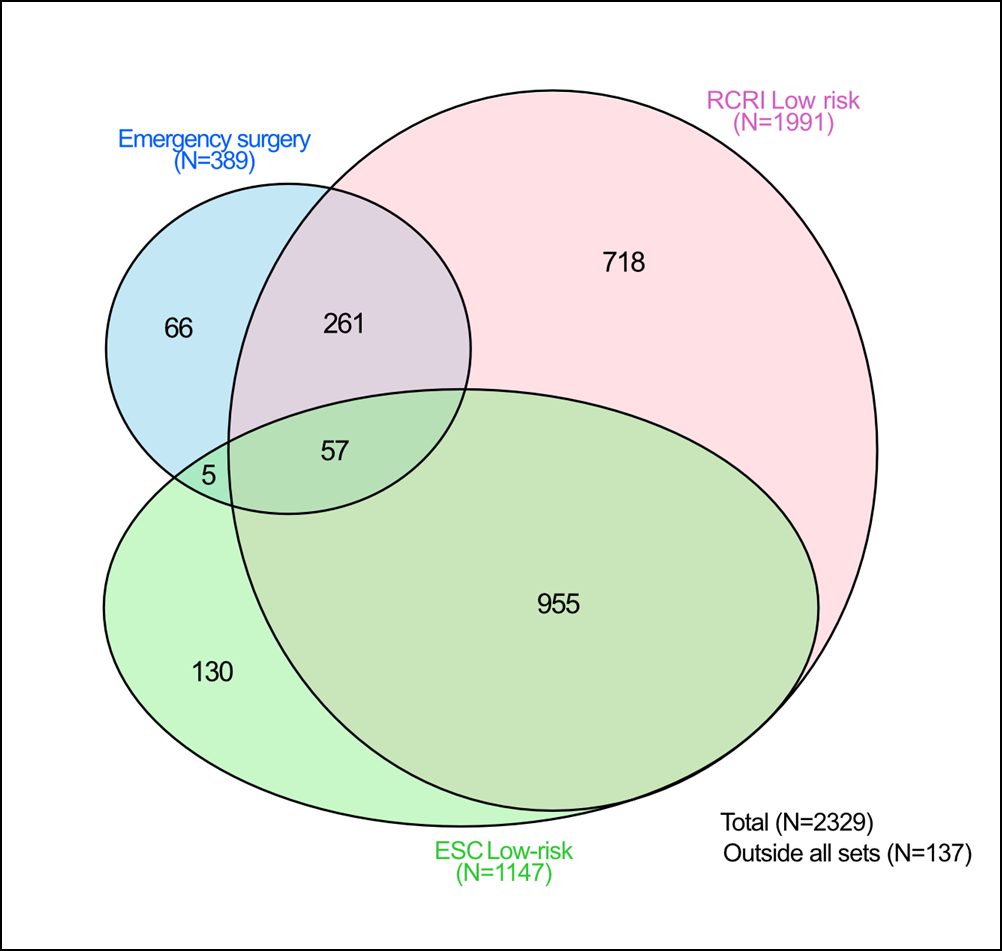


**Figure S6.** The representative case with abnormal coronary angiography (CAG) results requiring revascularization before proceeding to surgery. (A) ECG and AI-ECG results before CAG; (B) the closest ones before surgery; (C) CAG findings; (D) CAG after PCI.

**
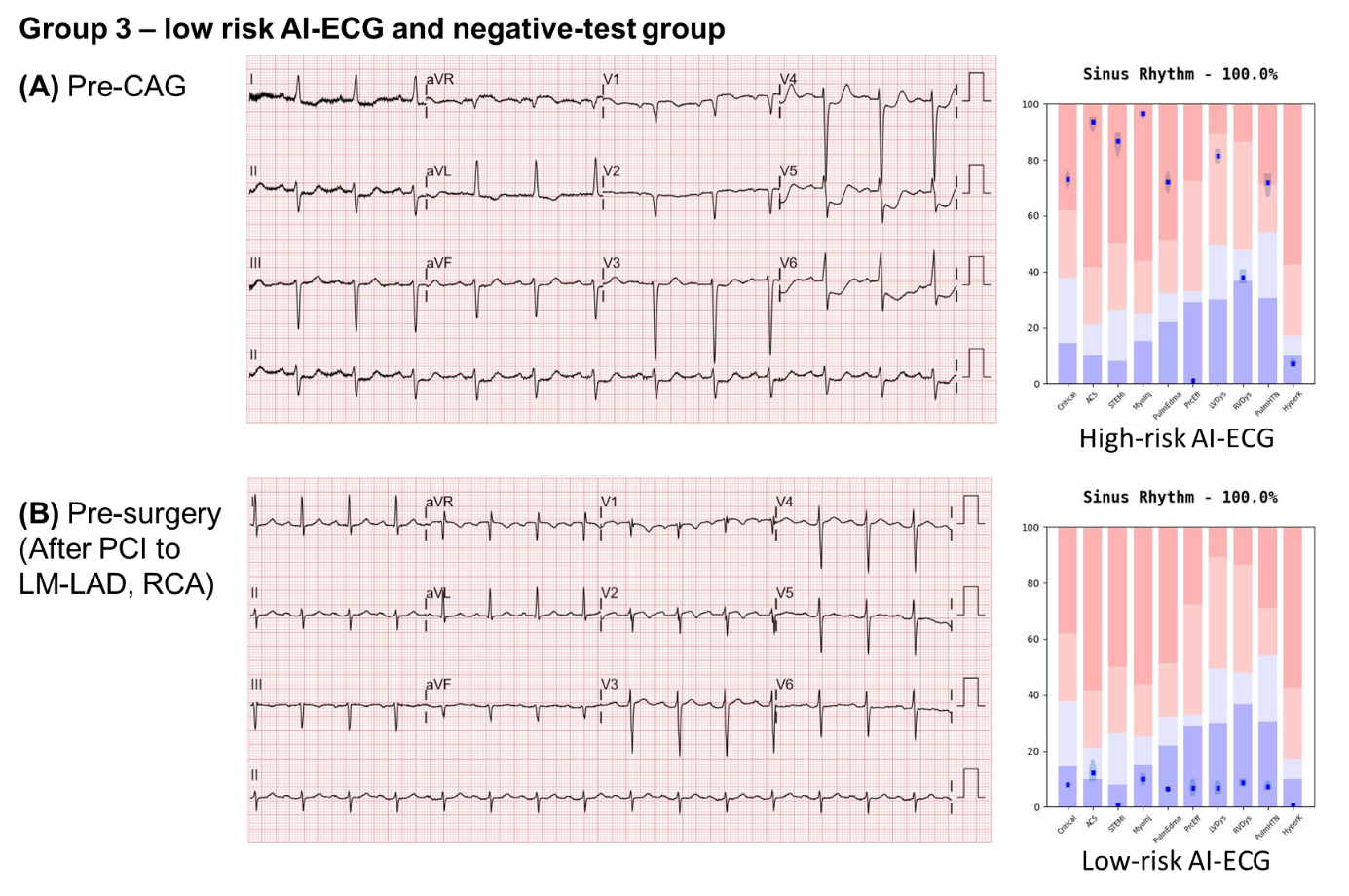
**


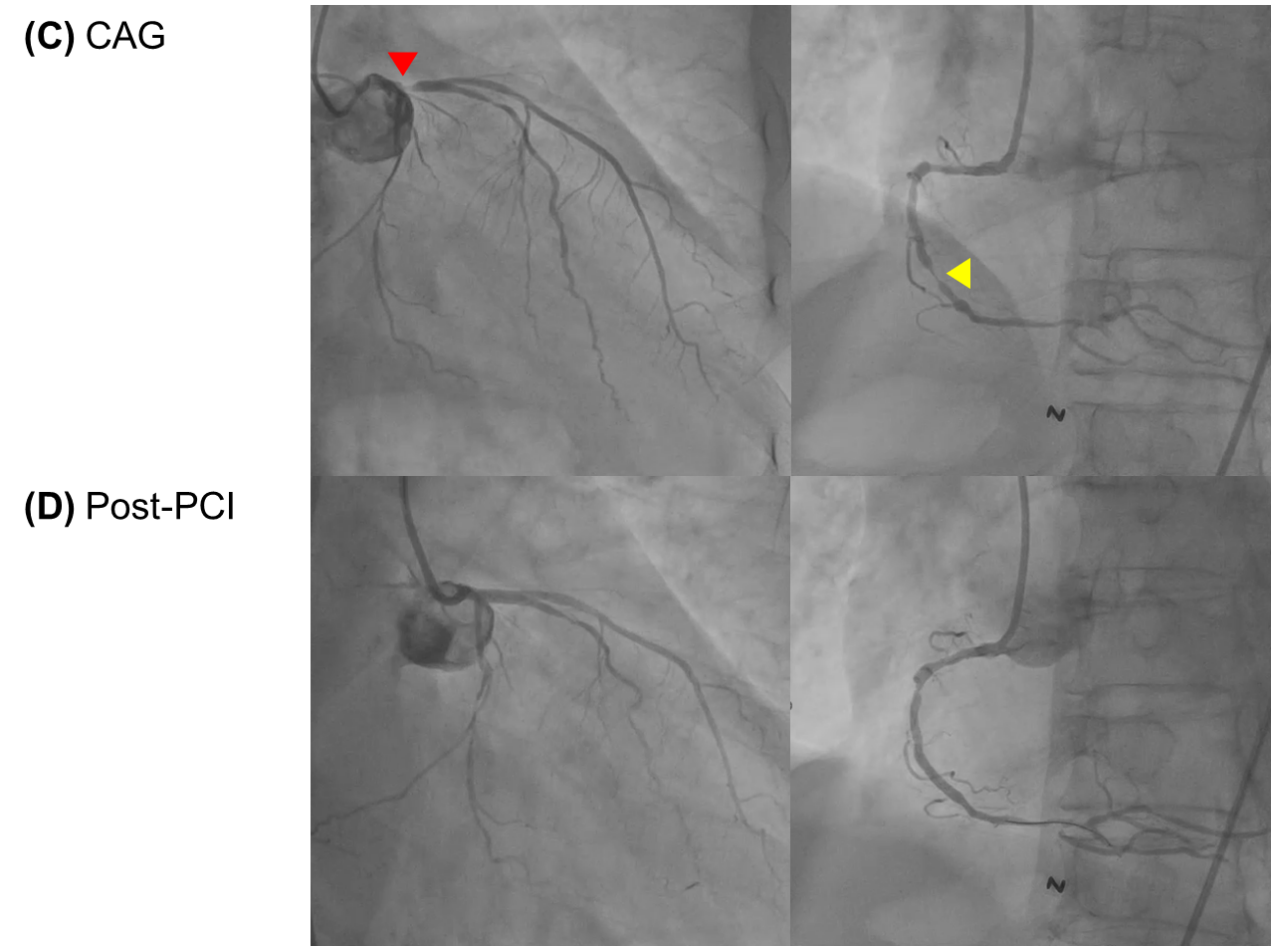

Supplement: Multimedia Appendix 1 [file jmir_v28i1e90099_app1.docx]
